# Metabolic stress constrains microbial L-cysteine production in *Escherichia coli* by accelerating transposition through mobile genetic elements

**DOI:** 10.1186/s12934-023-02021-5

**Published:** 2023-01-16

**Authors:** Kevin Heieck, Nathanael David Arnold, Thomas Bartholomäus Brück

**Affiliations:** Technische Universität München, Lichtenbergstraße 4, 85748 Garching, Germany

**Keywords:** L-cysteine, *E. coli*, Minimal genome strain, Metabolic stress, Insertion sequences, Transcriptomics, Deep sequencing, Metabolic engineering

## Abstract

**Background:**

L-cysteine is an essential chemical building block in the pharmaceutical-, cosmetic-, food and agricultural sector. Conventionally, L-cysteine production relies on the conversion of keratinous biomass mediated by hydrochloric acid. Today, fermentative production based on recombinant *E. coli*, where L-cysteine production is streamlined and facilitated by synthetic plasmid constructs, is an alternative process at industrial scale. However, metabolic stress and the resulting production escape mechanisms in evolving populations are severely limiting factors during industrial biomanufacturing. We emulate high generation numbers typically reached in industrial fermentation processes with *Escherichia coli* harbouring L-cysteine production plasmid constructs*.* So far no genotypic and phenotypic alterations in early and late L-cysteine producing *E. coli* populations have been studied.

**Results:**

In a comparative experimental design, the *E. coli* K12 production strain W3110 and the reduced genome strain MDS42, almost free of insertion sequences, were used as hosts. Data indicates that W3110 populations acquire growth fitness at the expense of L-cysteine productivity within 60 generations, while production in MDS42 populations remains stable. For the first time, the negative impact of predominantly insertion sequence family 3 and 5 transposases on L-cysteine production is reported, by combining differential transcriptome analysis with NGS based deep plasmid sequencing. Furthermore, metabolic clustering of differentially expressed genes supports the hypothesis, that metabolic stress induces rapid propagation of plasmid rearrangements, leading to reduced L-cysteine yields in evolving populations over industrial fermentation time scales.

**Conclusion:**

The results of this study implicate how selective deletion of insertion sequence families could be a new route for improving industrial L-cysteine or even general amino acid production using recombinant *E. coli* hosts. Instead of using minimal genome strains, a selective deletion of certain IS families could offer the benefits of adaptive laboratory evolution (ALE) while maintaining enhanced L-cysteine production stability.

**Supplementary Information:**

The online version contains supplementary material available at 10.1186/s12934-023-02021-5.

## Background

The amino acid L-cysteine, harbouring a thiol group, provides a high redox activity in cell metabolism, plays a crucial role in protein folding, functions as a catalytic residue of several enzymes and serves as a building block of 5-L-glutamyl-L-cysteinylglycine (GSH) and as a donor compound of sulphur, which is required for the synthesis of Fe/S clusters, biotin, coenzyme A and thiamine [1, 2].

Besides the essential function in metabolism, L-cysteine is also of considerable industrial importance, with applications ranging from pharmaceutical products and cosmetics over food production to feed additives in livestock farming [3, 4].

To date, the cheapest and thereby most prevalent means of L-cysteine production involves chemical hydrolysis of—and extraction from—keratinous biomass, such as feathers, pig bristles and animal hair by means of electrolysis [5]. Up to 27 tons of hydrochloric acid are required to obtain 100 kg of a racemic mixture of cysteine from 1.000 kg raw material [5, 6]. In order to circumvent negative impacts upon the environment associated with hydrochloric waste disposal, alternative technologies such as fermentation and enzymatic conversion have been explored and rapidly gained significance since their implementation. In 2004, 12% of the globally manufactured L-cysteine global originated from fermentation [7].

The enzymatic conversion of DL-2-amino-∆2-thiazoline-4-carboxylic acid (D-ATC) to L-cysteine with *Pseudomonas* spp. derived enzymes is limited by product inhibition [8, 9]. For biotechnological L-cysteine production, the bacteria *C. glutamicum* and *E. coli* harbouring optimised plasmids represent the dominant expression organisms. Since titres from *C. glutamicum* are low (approx. 950 mg/L), *E. coli* is the preferred host for L-cysteine production by fermentation [10].

However, there are still major obstacles in upscaling fermentation processes with engineered microorganisms. The stability of strains with synthetic production is highly fragile and presents a challenge when implementing bioprocesses on a large scale [11, 12]. The introduction of designed plasmid constructs and the upregulation of the genetic elements for recombinant L-cysteine production pose a defiance to the tightly regulated homeostasis within host cells [13, 14]. This metabolic load hinders the expression of other genes, thereby negatively affecting growth rate and promoting evolutionary pressure [15–17]. Furthermore, L-cysteine has an inhibitory to toxic effect on *E. coli* cell growth, depending on the concentration present in cells [18]. In microorganisms, several concepts are reported that can lead to a selection advantage and thus to both phenotypic and genotypic variation within populations [19, 20]. In bacteria, activation of mobile genetic elements, such as insertion sequences (IS) and corresponding transposons can lead to mutagenesis-based inactivation of synthetic constructs [21]. In addition, expression of regulatory elements of the SOS response has been shown to increase the expression of error-prone DNA polymerases, which can indirectly induce further mutations in recombinant gene elements, such as plasmids [22, 23]. These effects have a negative impact on the time dependent-productivity (space–time yield) and consequently the total yield of the biomolecule to be produced. This observation is especially relevant in continuous fermentations, where populations reach high generation numbers while being exposed to prolonged metabolic stress [24, 25].

**Fig. 1:**
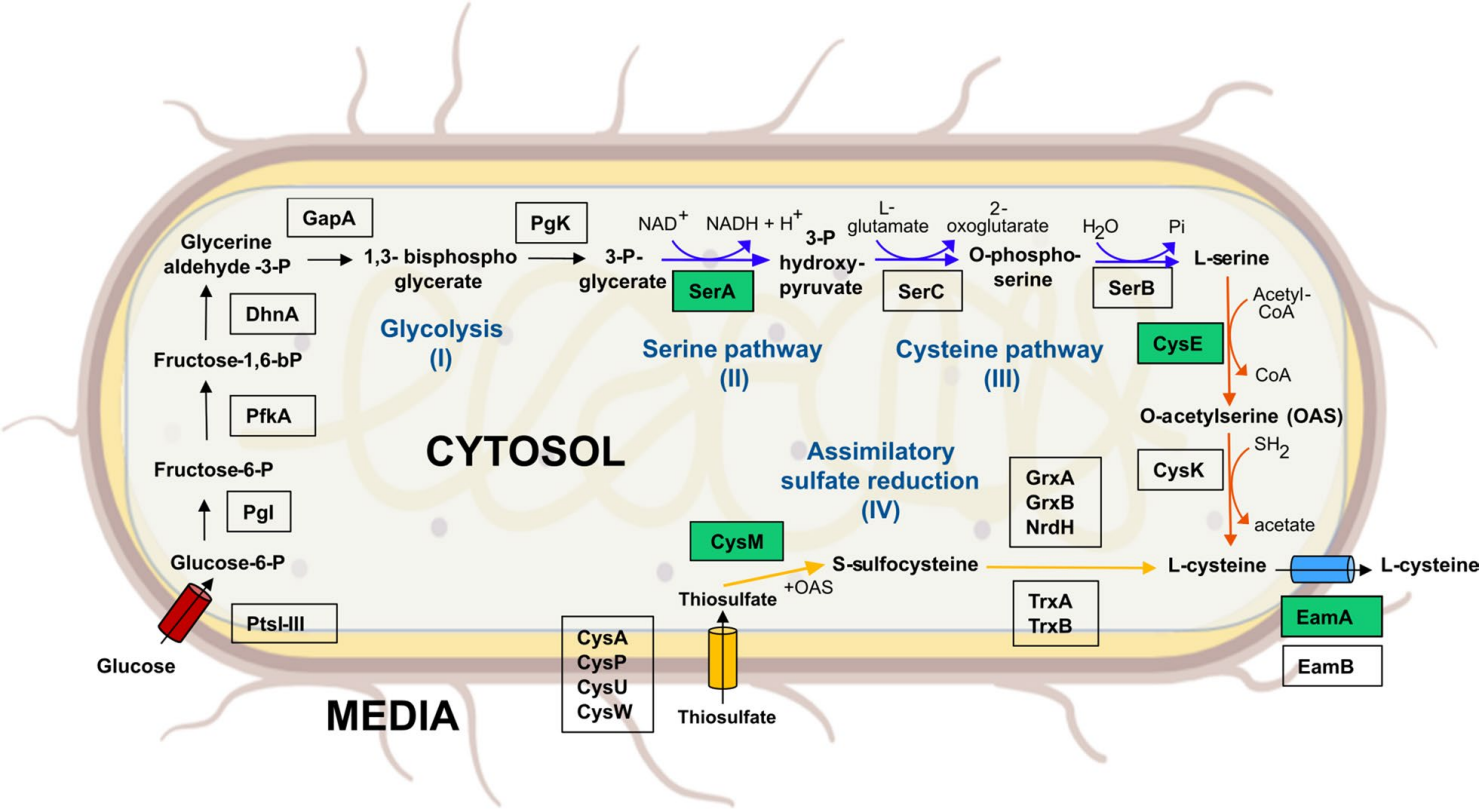
Metabolic engineering strategies for L-cysteine biosynthesis in *Escherichia coli*: Through glycolysis (I, black arrows), the essential intermediate 3-phopsho-glycerate is produced, which is incorporated into L-serine biosynthesis (II, blue arrows). As a precursor amino acid, L-serine is used for the formation of L-cysteine (III, orange arrows). Alternatively, L-cysteine can be synthesised by the assimilatory sulphate reduction pathway (IV, yellow arrows). L-cysteine is transported out of the cell. Corresponding proteins for conversion of substrates are shown in black boxes. Boxes with green background represent expressed proteins within the synthetic plasmid constructs in this work. *PtsI-III* Phosphotransferase system I-III, *PgI* Glucose-6-phosphate isomerase, *PfkA* Phosphofructokinase 1, *DhnA* Fructose-bisphosphate aldolase, *GapA* Glyceraldehyde-3-phosphate dehydrogenase A, *PgK* Phosphoglycerate kinase, *SerA* D-3-phosphoglycerate dehydrogenase, *SerC* Phosphoserine aminotransferase, *SerB* Phosphoserine phosphatase, *CysE* Serine acetyltransferase, *CysK* Cysteine synthase A, *CysA,P,U,W* ATP-dependent sulphate/thiosulphate uptake system, *CysM* Cysteine synthase B, *GrxA,B* Glutaredoxin 1,2, *NrdH* Glutaredoxin-like protein, *TrxAB* Thioredoxin 1,2, *EamA,B* Cysteine exporter. Created with BioRender.com

Metabolic engineering of *E. coli* strains optimising L-cysteine production, targets the overexpression of specific bottleneck genes (Fig. 1). In order to capture 3-P-glycerate from glycolysis and feed it into the synthesis of the precursor amino acid L-serine to finally convert it to L-cysteine, the two feedback-resistant genes *serA* and *cysE* are overexpressed [26, 27]. An L-cysteine production pathway uncoupled from glycolysis involves the assimilatory reduction of sulphate. With the expression of *cysM*, assimilated thiosulphate is converted to L-cysteine via an intermediate step. Since large amounts of L-cysteine have an inhibitory growth effect on *E. coli* cells, the overexpression of an L-cysteine efflux gene (*eamA*) is essential [28].

The aim of this study was to investigate the genotypic and phenotypic alterations of engineered L-cysteine producing K-12 MG1655 derived *E. coli* strains leading to L-cysteine production decline. For the first time, a combined omics-approach was applied in order to unravel the underlying mechanisms within an industrially relevant L-cysteine production system. Therefore, the *E. coli* production strain W3110 and a reduced genome strain (MDS42) free of transposable elements were selected. By simulating long-term cultivations in shaking flasks, we achieve generation numbers (> 60) of industrial fermentation processes. In accordance with our hypothesis, a L-cysteine productivity collapse of up to 85% could be detected on a laboratory scale. Using differential transcriptomics, we uncover strong shifts in L-cysteine and sulphur stress related transcripts between early and late populations. Furthermore, Illumina-based plasmid deep sequencing data strongly suggested that predominantly IS3 and 5 family transposases induce rapid plasmid rearrangements. Both observations, presumably in combination, disrupt L-cysteine production in late W3110 populations while L-cysteine production in MDS42 populations remain stable, further supporting the involvement of insertion sequences.

## Results and discussion

### Stability of L-cysteine-producing phenotypes

**Fig. 2:**
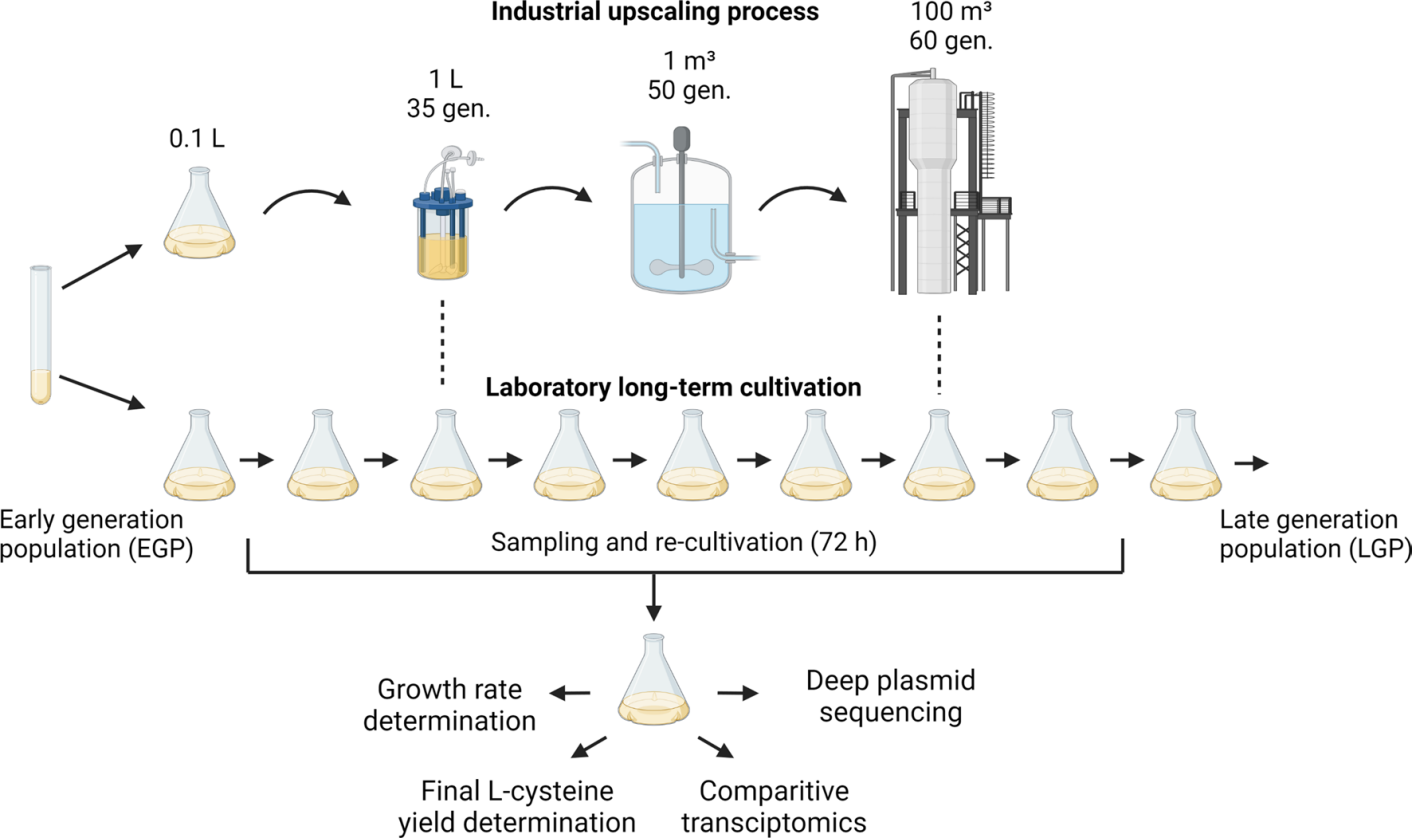
Laboratory long-term cultivation of L-cysteine producing *E. coli* cells. Adapted from Rugbjerg et al., 2018. One “wild-type” *E. coli* MG1655 strain (W3110) and a reduced genome strain (MDS42) were cultivated harbouring one out of three plasmid constructs (Fig. 1A). By serially transferring samples every 10 or 5 h into fresh medium, respectively, we imitate generation numbers found in large-scale industrial bioreactors. Thereby the cultures were kept in the exponential phase throughout the experiment. Sample collection was continued until desired generation numbers (> 60) were achieved. Subsequently, all cryo-samples were re-cultivated for 72 h during which growth rate- and final L-cysteine yields were monitored. RNA extractions for comparative transcriptomics and plasmid extractions for deep plasmid sequencing were performed on the early and late generation population (EGP, LGP) samples for each strain and plasmid construct, respectively. Created with BioRender.com

To study phenotypic and genetic diversities in L-cysteine producing *E. coli* populations over timescales relevant on industrial levels, we simulated a gradual scale-up growth process (Fig. 2).

**Fig. 3:**
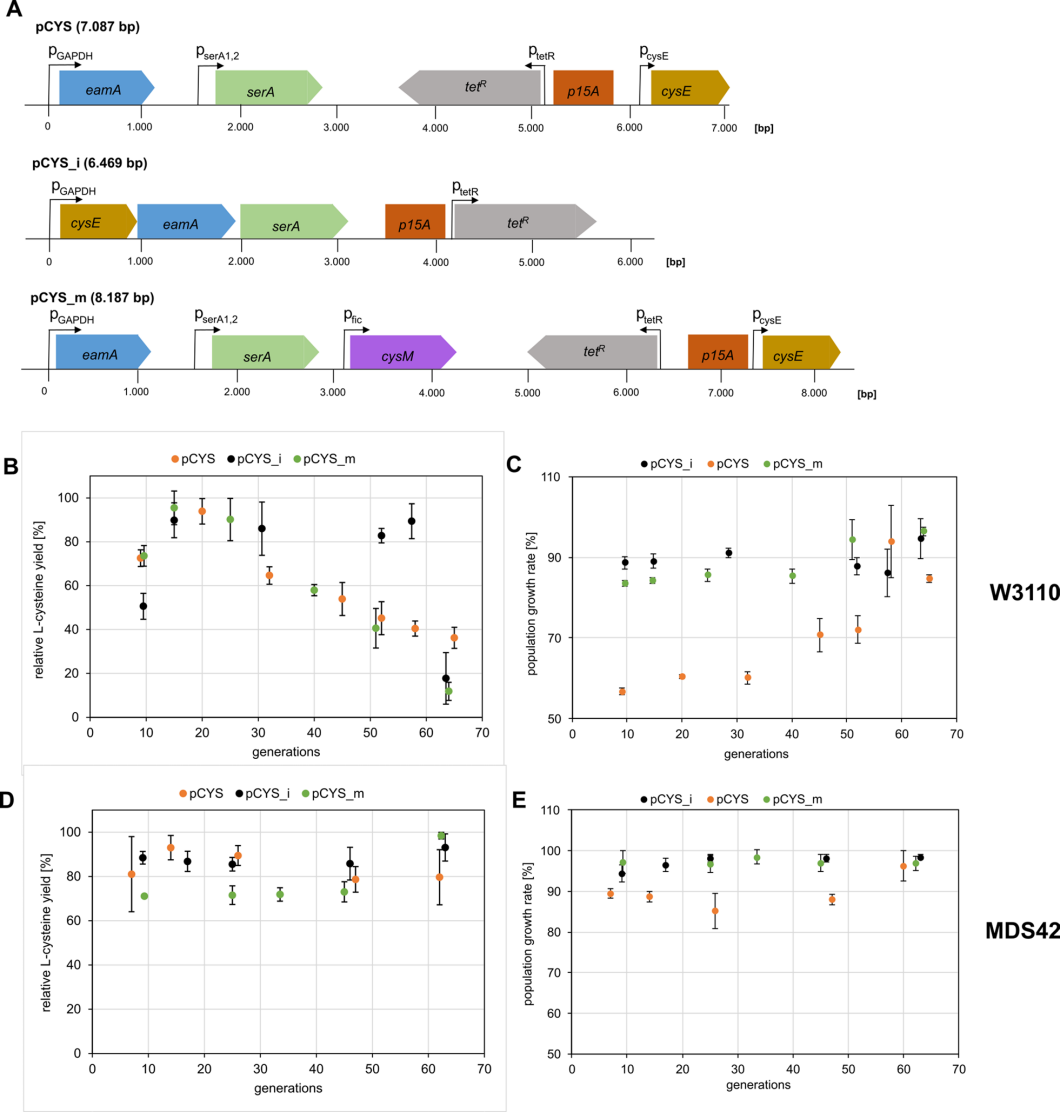
Stability of L-cysteine producing phenotypes. **A**: Plasmids used for transformation in *E. coli* W3110 and MDS42 to study the stability of L-cysteine production. Boxed arrows indicate genes located on the plasmid, whereas bended arrows display corresponding promoters. **B-E**: Plots of relative L-cysteine yields in % (B + D) and the population growth rates (C + E) as a function of the number of accumulated generations. Cultivation and subsequent L-cysteine yield measurements were carried out in biological triplicates of W3110 and MDS42 with the three different plasmids shown in Fig. 3A. L-cysteine yields were normalised based on the OD_600_ and the highest yield of the corresponding biological replicate (Additional file 1: fig. S1). Population growth rates were normalised based on growth rates of the corresponding non-producing strains harbouring empty vectors (Additional file 1: table S3)

We cultivated the minimal genome strain MDS42, almost free of any insertion sequences (IS), next to the traditional K-12 strain W3110 to determine potential effects of transposable elements that disrupt synthetic constructs. Specifically, we cultivated three different clones of two *E. coli* strains, each with an L-cysteine producing plasmid (Fig. 3A). The original plasmid pCYS possessed bottleneck genes for L-cysteine biosynthesis with the corresponding genomic promotor regions (Fig. 3A). CysE and SerA are feedback insensitive variants as described in references [26, 27, 29, 30]. For cloning of pCYS_i the genes were arranged in one operon with GAPDH as a constitutive promotor. Furthermore, non-coding backbone sequences were trimmed to potentially alleviate metabolic burden on production cells. The plasmid pCYS_m carries the additional gene *cysM*, coding for cysteine synthase B, under the control of the stationary phase promoter p*fic*. This way, L-cysteine production should be shifted to the stationary phase, therefore temporally uncoupling cell growth from L-cysteine biosynthesis during the 72 h re-cultivations.

In order to prevent plasmid loss, we kept the cultures at a constant antibiotic selection. By serially transferring the W3110 production strains every 10 h and the MDS42 production strains every 5 h (due to its higher growth rate), we achieved populations > 60 cell generations (Additional file 1: Table S2). Hence, an exponential growth phase was guaranteed at all times throughout the experiment. At each transfer step, we sampled the growing *E. coli* populations and immediately cryopreserved them, in order to study time dependent phenotypic and genetic variability following a 72 h re-cultivation of the respective culture samples.

Final L-cysteine yields of each sampled population were determined throughout the simulated long-term cultivation. While an initial increase in relative L-cysteine yields of transformed W3110 strains towards a maximum was measured, the yields at 60 accumulated generations only reached 15–35% of the maximal product concentration. The cysteine yields of the W3110 strain with integrated pCYS_i plasmid remained at a constant high level until a drop at 63 generations, while the other plasmids showed a steady decline from generation 15–20 onwards (Fig. 3B). Transformed MDS42 strains showed no steady decline in generated L-cysteine yields with accumulating generations. Instead, the L-cysteine space time yields were stable at 75–100% (Fig. 3D). However, total L-cysteine yields in MDS42 populations were overall lower compared to W3110 populations (Additional file 1: Fig.S1). In case of the MDS42 strain, it should be taken into account that due to the deletion of 704 genes physiological differences exist in comparison to the W3110 strain. The effects depend on the biomolecule to be produced, when employing MDS42 as a cell factory.

Furthermore, we tested strain viability by determining growth rates of the sampled populations. As a function of generation numbers, growth rates of W3110 differed depending on the transformed plasmid (Fig. 3C). W3110 populations with integrated pCYS_i plasmid showed no considerable changes in growth rate. In contrast, we observed a substantial change of fitness in case of W3110 with integrated pCYS_m and pCYS. When comparing initial and final growth rates, we detected an increase of 13% for pCYS_m, and 27% for pCYS. There was no considerable effect on fitness in transformed MDS42 populations (Fig. 3D).

Overall, the decline in L-cysteine yields tended to correlate with the increase in growth rates in an inversely proportional pattern, indicating an acquisition of fitness at the expense of L-cysteine production.

### Transcriptome analyses reveals strong differences regarding expression levels in W3110 populations and few differences in MDS42 populations over the course of long-term cultivation

**Fig. 4:**
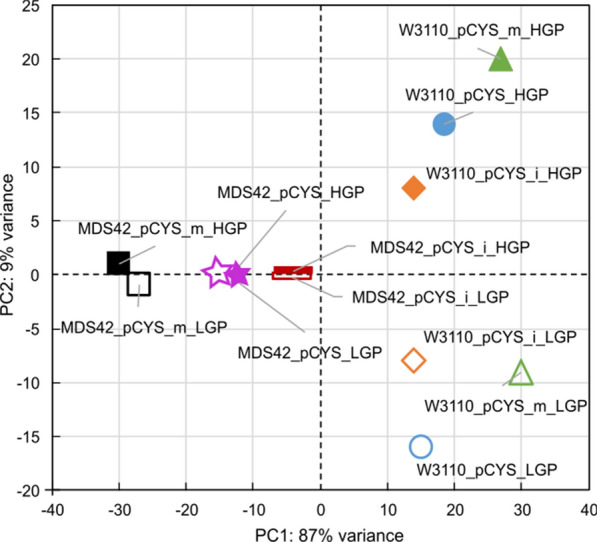
Principal component plot of all samples subjected to transcriptome analysis. Later generation populations (LGPs) are marked with filled-in shapes, while shapes of early generation populations (EGPs) are unfilled. LGPs correspond to populations with 60–65 evolved generations, while EGPs correspond to populations with 7–10 evolved generations. Related samples are marked with the same colour. Samples displayed in close proximity indicate very few differences between them while samples placed more distantly suggest bigger variations

To determine potential causes for the collapse of L-cysteine production, we examined the transcriptomes of W3110 and MDS42 populations subjected to the long-term cultivation. We sequenced and compared transcriptomes from early generation populations (EGP) with later generation populations (LGP). Interestingly, when assessing global relationships between samples, we found that EGPs and LGPs of the MDS42 strains hardly differed from each other, while there were larger deviations in W3110 populations (Fig. 4). This result is consistent with previous findings of a limited evolutionary adaptability of the MDS42 strain and illustrates why we observed negligible changes in L-cysteine production and fitness in MDS42 populations [31, 32].

### Metabolic clustering helps understanding the burden on cell populations during L-cysteine production

**Fig. 5:**
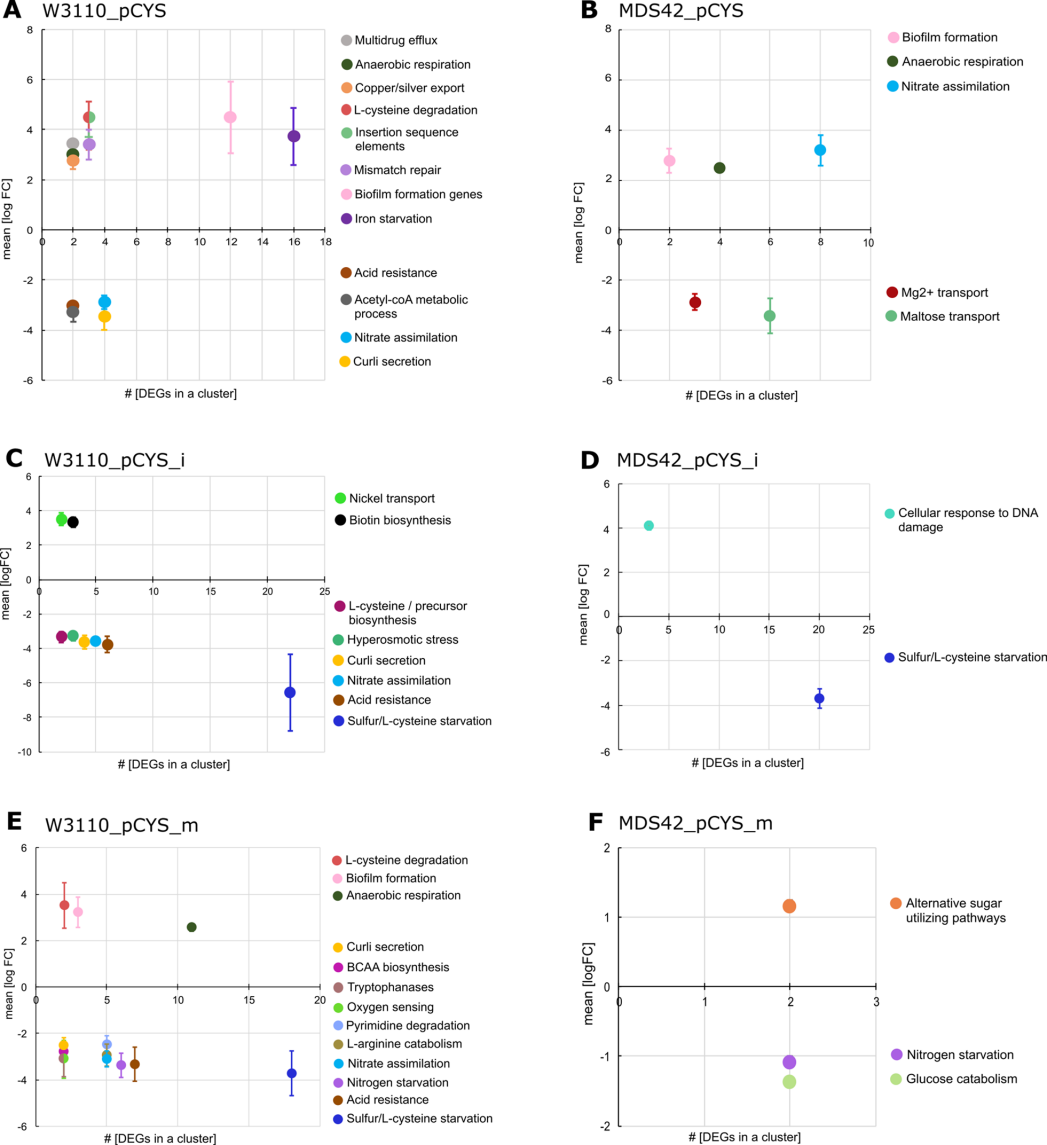
Clustering of differentially expressed genes (DEGs) based on the metabolic function in *E. coli*. For this, logarithmic fold change (logFC) medians of DEGs with the same metabolic function were plotted against the number of DEGs within this group. Fold changes were calculated by dividing values of the later generation population (LGP) by values of the early generation population (EGP). LGPs correspond to populations with 60–65 evolved generations, while EGPs correspond to populations with 7–10 evolved generations. *E. coli* strains W3110 (A, C, E) and MDS42 (B, D, F) transformed with one of three plasmids engineered for L-cysteine production (pCYS: A, B; pCYS_i: C, D; pCYS_m: E, F) were analysed. A p-value cut-off < 0.05 was selected. Genes with unknown function as well as clusters mapped with only one gene were disregarded here, but can be found in Additional file 1: Tables S6-S11. **A**: 81 genes in total (57 genes within 12 cluster, 20 genes with unknown function and 4 single gene clusters). **B**: 34 genes in total (34 genes within 5 cluster, 6 genes with unknown function and 2 single gene clusters). **C**: 65 genes in total (47 genes within 8 cluster, 10 genes with unknown function and 8 single gene clusters). **D**: 23 genes within 2 cluster. **E**: 105 genes in total (70 genes within 12 cluster, 17 genes with unknown function and 18 single gene clusters. **F**: 14 genes in total (6 genes within 3 cluster, 4 genes with unknown function and 4 single gene clusters)

To gain a deeper insight into the differences in expression levels in W3110 and MDS42 populations, we examined differentially expressed genes (DEGs) with a p-value cut-off < 0.05 (Additional file 1: Tables S6–S11). After clustering the DEGs according to their metabolic function, we subsequently plotted the number of DEGs in a cluster against the mean fold change of those in the cluster (Fig. 5). Overall, we noticed a lower number of different metabolic clusters in MDS42 populations than in W3110 populations. In general, stress features such as nitrogen starvation, acetyl-coA-, nitrate-, and carbon assimilation were up-regulated in EGPs, indicating a resource-draining L-cysteine production process. However, most up-regulated features in EGPs, where L-cysteine production was highest, belonged to the cluster of sulphur and L-cysteine starvation (Table 1). The metabolic flux towards L-cysteine coupled with overexpression of its corresponding exporter gene *eamA* most likely caused the intracellular deficiency of sulphur and the sulphuric amino acid.

**Table 1:** List of sulphur and L-cysteine metabolism related operons detected as clusters in transcriptome analysis

DEGs of sulphur/ L-cysteine metabolism related operons	Metabolic function	Literature
*cyuAP*	L-cysteine desulfidase, L-cysteine utilization permease	[35], [36]
*aslB*	Putative anaerobic sulfatase maturation enzyme	[37]
*tauABCD*	Taurine utilization proteins	[38]
*ssuEADCB*	Aliphatic sulfonates utilization proteins	[39]
*cysPUWA, sbp*	Sulphate/thiosulphate transport proteins	[40], [41]
*cysDNC*	Sulphate activation proteins	[42]
*cysJIH*	Sulphite reductase proteins	[43]
*tsuAB*	Thiosulphate transport proteins	[44]

In LGPs, additionally to up-regulated clusters such as biofilm formation, iron starvation and anaerobic respiration, we also identified features belonging to an L-cysteine degradation operon (Table 2). The corresponding D/L-serine membrane transporter CyuP was potentially protecting the cell from increasing sulphur starvation by shuttling serine out of the cell before this precursor amino acid was further metabolised to L-cysteine. CyuA, on the other hand, may have acted as an L-cysteine desulfidase releasing sulphur by degradation for cell homeostasis. The observation of the decrease in measured L-cysteine yields in LGPs supports this hypothesis. Furthermore, we suspected that the expression of the *cyuAP* operon was triggered by sulphur starvation.

**Table 2:** List of genes related to genetic instability detected in transcriptome analysis

DEGs belonging to genetic instability	Metabolic function	Literature
*insJK*	IS3 family transposase	[45]
*yjgZ*	Putative IS66 family transposase	InterPro
*ybcN, rusA, iprA*	DNA repair	[46], [47], [48]

Additionally, it would be worth to consider adding the transcription factor *cysB* into the plasmid construct, therefore enhancing sulphur utilisation and sulfonate-sulfur catabolism via L-cysteine production. Recombinant expression of *cysB* has been demonstrated to enhance L-cysteine yields in *E. coli* [33].

In the further course of the transcriptome analysis, we found clusters that indicated genetic instability of the production strains. Transposases for insertion sequence families 3 and 66 as well as mismatch repair genes were upregulated in LGPs (Table 2). In the sulphur and L-cysteine starvation cluster of EGPs we detected *cysD*, a gene that facilitates stress-induced mutagenesis (SIM) [34]. Therefore, we examined the impact of this genetic instability on the plasmid level.

### Deep sequencing reveals an accumulation of insertion sequences (IS) in production plasmids of evolving populations

**Fig. 6:**
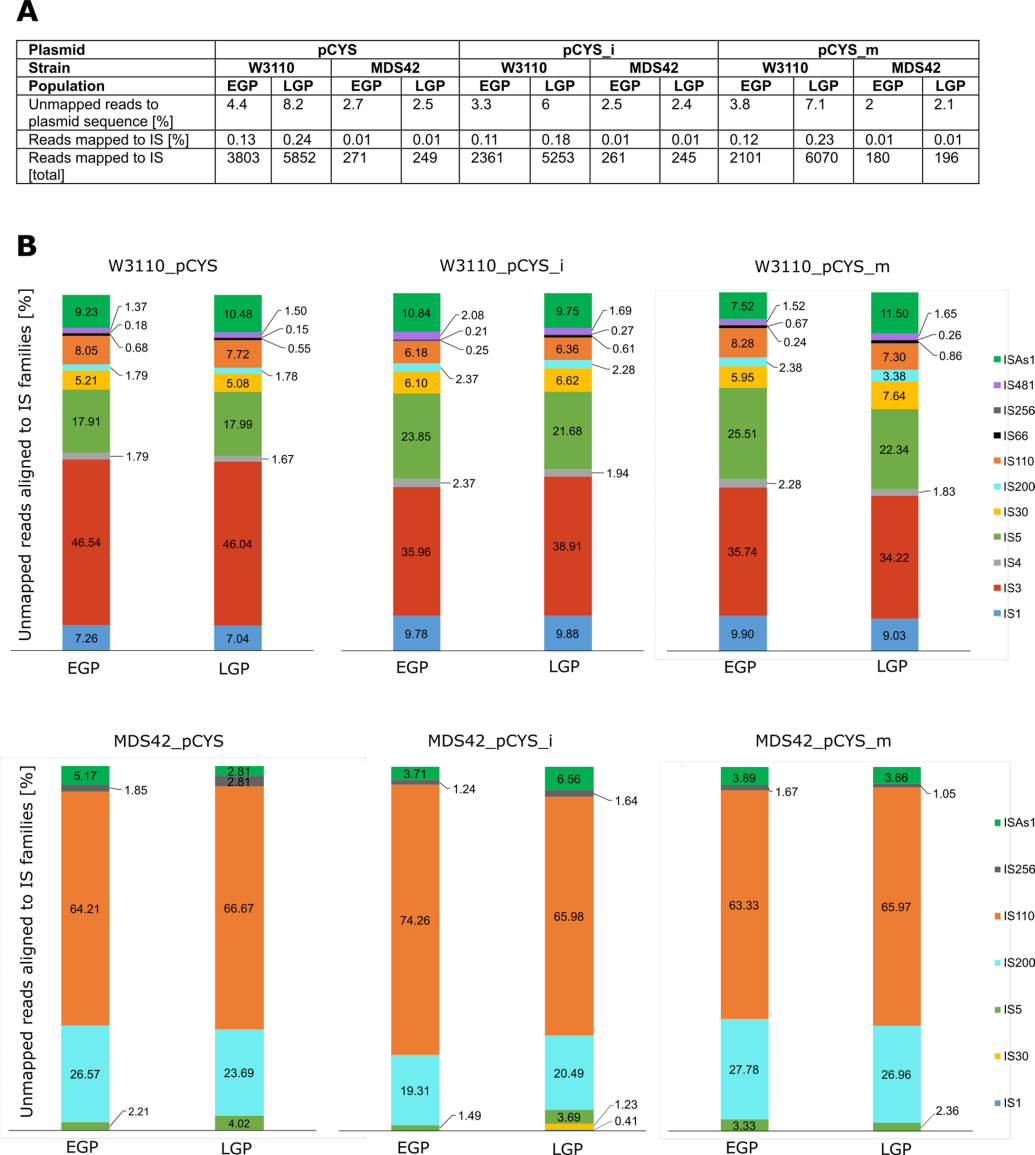
Mapping of sequenced reads against insertion sequence (IS) families. **A**: Table showing the percentages of reads that did not map to the plasmid sequences as well as the percentages of those unmapped reads mapped to IS. Plasmids pCYS, pCYS_i and pCYS_m extracted from early and late generation populations of W3110 and MDS42 got deep sequenced (Methods). **B**: Proportions of unmapped reads aligned to the different IS families in percent. The caption is arranged in the same order as the stacked bars

When calculating and comparing the contents of extracted plasmids per µg cells of EGPs and LGPs, we observed no relevant differences (Additional file 1: Table S12). The L-cysteine production decline could therefore not be linked to lower plasmid quantities in LGPs. In a next step, we deep sequenced plasmids from EGPs and LPGs and mapped the sequenced reads against the associated plasmid sequence reference (Methods). The average per base coverage depth was > 140.000x (Additional file 1: Fig. S2–S4). Consistent with other studies, we did not find genetic variance in plasmid genes by single nucleotide polymorphism (SNP) analyses (Additional file 1: Table. S13), [20, 21]. Interestingly, we noticed an increase in unmapped reads in LGPs of W3110 compared to EGPs (Fig. 6A). In a next step, we mapped these reads against an insertion sequence database from *E. coli* revealing higher coverages of aligned reads to IS in LGPs (Fig. 6A). Surprisingly, we also identified reads, that could be mapped to IS in plasmids from MDS42 populations, although the number was reduced by tenfold. In order to assign the mapped reads to the different IS families, we used NCBI’s megablast algorithm (Methods).

The distribution of the different IS families was very similar for all three production plasmids. This observation was the same for EGPs compared to LGPs (Fig. 6B). Nevertheless, the total number of reads that could be mapped to IS doubled on average when comparing W3110 EGPs with LGPs, while the number remained the same for MDS42 populations. This indicates, that transpositions of different IS families propagated in similar frequencies. For plasmids extracted from W3110 populations, we most frequently identified reads that could be mapped to the IS3 and IS5 family, which supports the result of a high expression of *insJK*, an IS3 family transposase, in LGPs of W3110_pCYS. In addition, reads mapped to the ISAs1 family were very abundant. Among plasmids from MDS42 populations, the reads could be mapped mainly to sequences belonging to IS200- and IS110 families. Due to deletion of most IS, the few transposition events in MDS42 populations are in line with the stable L-cysteine production and growth rates, rendering *E. coli* MDS42 as a stable host for industrial L-cysteine fermentations.

## Conclusions

The data in this study suggest that insertion sequence (IS) transposition, triggered by the metabolic stress of L-cysteine production, leads to structural genetic rearrangements in production plasmids. Within 60 generations, as achieved in industrial large-scale fermentations, L-cysteine production capacities collapsed by up to 65–85% in *E. coli* W3110 populations while growth rates increased up to 27% depending on the L-cysteine plasmid construct. Contrarily, *E. coli* MDS42 populations exhibited nearly stable space–time yields of 75–100% with no considerable effect on growth related fitness.

Metabolic stress of L-cysteine producing populations, cultivated in shake flasks, as illustrated in this study, certainly differs from cultures grown in large-scale bioreactors, e.g. O_2_ limitation as reflected in upregulation of anaerobic genes in LGPs. Yet the fundamental issue is the same: Cells are driven to eliminate factors that impair growth fitness by evolutionary adaptation. Frequently, these factors involve metabolically burdensome compounds meant to be produced by engineered *E. coli* cells, establishing non-producing populations in continuous cultivations [21, 49–52].

Common industrial practice employs clone banks of production strains stored as frozen aliquots. Although the population appears inconspicuous for a number of divisions, a few cells present in the starting seed may harbour genetic mutations. Cell bank aliquots with rare pre-existing mutations that disrupt production, should therefore be screened beforehand, using deep-sequencing techniques.

Opposed to previous work relying on PCR-based methods for IS identification, we chose a deep sequencing approach on population level circumventing amplification biases [53]. Thereby, an accumulation of predominantly IS3 and IS5 fragments could be detected in W3110 LGPs, whereas IS accumulation was neglectable in MDS42 populations. However, due to read length limitations of the Illumina sequencing technology, mapping of IS to the corresponding plasmid reference sequences pose a hindrance. In addition, repetitive sequences within IS complicate distinguishability and read assembly. For this purpose, the Burrows-Wheeler aligner’s Smith-Waterman alignment (BWA-SW) is suitable to capture chimeric reads containing junctions between plasmid and IS sequences with frequent alignment gaps. From a future perspective, we suggest the usage of long read sequencers such as PacBio or Oxford Nanopore for a deeper understanding of IS mediated transposition into plasmid sequences. Full coverage of singular plasmid sequences would provide both the full length IS and its insertion site.

Consequently, the genetically stable minimal genome *E. coli* strain MDS42 rendered as a promising host for L-cysteine production. However, MDS42, while showing a superior growth rate and stability compared to W3110, exhibits an overall lower L-cysteine space–time yield (Additional file 1: Fig. S1).

This study postulates that selective deletion of IS and the corresponding transposases in genomes of industrially relevant production strains, such as W3110 can be used as a new, more-targeted approach to optimise the *E. coli* based industrial L-cysteine production. This could provide a trade-off between increased plasmid stability over the entire production phase while maintaining evolutionary adaptability of the cell system linked to high cell density fermentations at maximal growth rates and L-cysteine productivity.

## Materials and methods

### Strains and plasmids

Two parental *E. coli* K12 strains were used to construct the strains with three specific plasmids (Table 3):

**Table 3:** Plasmids used in this study: All plasmids are derived from pACYC184 with a p15A origin of replication and approx. 15 copies/cell

Plasmid	Relevant features	References
pCYS	p_GAPDH_:*ydeD*, p_serAp1,2_:*serA317*, p_cysE_: *cysE-XIV*, tet^R^, p15A	[26, 27, 29, 30]
pCYS_i	p_GAPDH_:*cysE-XIV-ydeD-serA317*, tet^R^ p15A	This study
pCYS_m	p_GAPDH_:*ydeD*, p_serAp1,2_:*serA317*, p_fic_:*cysM*, p_cysE_: *cysE-XIV*, tet^R^, p15A	This study

*E. coli* W3110: *F-* *λ- rph-1 INV(rrnD, rrnE).*

*E. coli* MDS42 (Scarab Genomics): MG1655 genome almost free from any ISs, *fhuACDB*, *endA* and more [48].

For introduction of the plasmids into the strains, standard transformations with chemical competent cells were performed. For the long-term cultivation experiments, single colonies of freshly transformed cells were used to inoculate precultures.

PCYS_i was generated by ligation of four amplified and digested amplicons in an equimolar ratio in a 40 µl reaction. The fragments contained the metabolic pathway genes of pCYS (*ydeD*, *cysE-XIV* and *serA317*) and the empty vector as a backbone. The digestions were conducted with SacI, XhoI, BamHI, PacI, and NcoI. Fast digest restriction enzymes, buffer, the T4 DNA Ligase as well as the Phusion DNA polymerase were used from Thermo Fisher Scientific. For amplification of different fragments, specific PCR primer were used (Additional file 1: Table S1). PCYS_m was generated by introducing the stationary phase promoter *pfic* and the *cysM* gene into the backbone of pCYS via Gibson cloning standard procedure (NEB). PCRs were performed with specifically designed primers (Additional file 1: Table S1).

### Media

A custom cultivation medium for the production of L-cysteine was used for all cultivations. This medium consisted of 10 g/L glucose, 5 g/L KH_2_PO_4_, 5 g/L (NH_4_)_2_SO_4_, 1 g/L Na_3_Citrat × 2 H_2_O, 0.9 g/L L-isoleucine, 0.6 g/L D, L-methionine, 0.5 g/L NaCl, 2 g/L ammonium thiosulphate, 1.2 g/L MgSO_4_ × 7 H_2_O, 18 mg/L thiamine-HCl, 9 mg/L pyridoxine–HCl, 15 mg/L tetracycline and 100 ml/L LB-medium. Additionally, 10 ml/L of a trace element solution was added. This trace element solution included 3.75 g/L H_3_BO_4_, 1.55 g/L CoCl_2_ × 6 H_2_O, 0.55 g/L CuSO_4_ × 5 H_2_O, 3.55 g/L MnCl_2_ × 4 H_2_O, 0.65 g/L ZnSO_4_ × 7 H_2_O, and 0.33 g/L Na_2_MoO_4_ × 2 H_2_O. The trace element solution is adjusted to pH 4.0 with HCl before autoclaving. Everything else got sterile filtrated after pH adjustment to 7–7.05. Tetracycline, vitamins, CaCl_2_ and MgSO_4_ were added immediately before inoculation.

### Simulated long-term cultivation

The simulated long-term cultivation method was adapted from Rugbjerg et al. [21]. Single colonies of freshly transformed cells were inoculated into 250 ml baffled shaking flasks containing 25 ml medium. W3110 strains were cultivated at 32 °C for 10 h and horizontal shaking at 150 r.p.m (New Brunswick Innova 44). Each strain harbouring one out of three plasmids got cultivated in triplicates. Due to a higher growth rate of the MDS42 strain, cultures were kept at 32 °C just for 5 h to always maintain an exponential growth phase. After each time point cultures were inoculated into 25 ml fresh medium (starting OD600 = 0.05) and incubated under the same conditions for another 10 h or 5 h. At each passage, sample’s OD_600_ were recorded to determine the accumulated generations (Additional file 1: Table S2) and 1 ml got snap-frozen with 1 ml 50% glycerol in liquid N2 and stored at − 80 °C. Subsequently the sampled cryo-cultures were re-cultivated for 72 h with a starting OD_600_ of 0.01 into 25 ml fresh medium under above mentioned conditions. Growth rates were monitored every 30 min. L-cysteine yield determination was performed after 72 h. RNA extraction was carried out at OD_600_ values of 0.6–0.8. Extraction of plasmid DNA was performed from the same culture (Fig. 2).

### Determination of L-cysteine yields by spectrophotometry

Each population sample from the glycerol stock was used for inoculation of 25 ml medium and the culture got cultivated at 32 °C at 150 r.p.m for 72 h (New Brunswick Innova 44). After incubation, 1 ml culture was centrifuged at 15.000 × g for 1 min. Both the pellet and the supernatant were further treated according to a modified method of Gaitonde to determine the L-cysteine yields (Additional file 1: Note 1) [54].

### Measurement of population growth rates

In order to measure population growth rates, the OD_600_ values of cultures grown for L-cysteine productivity analysis (as described in the previous section) were recorded every hour. Un-inoculated medium was used for background subtraction. Calculation of growth rates were performed with the following formula:

$$r= {\frac{N(t)}{N(0)}}^\frac{1}{t}-1$$

where: N(t): cell number at time t, N (0): cell number at time 0, r: growth rate and t: time passed. Values of measured growth rates can be found in Additional file 1: Table S3. Population growth rates were normalised based on growth rates of the corresponding non-producing strains harbouring empty vectors.

Cell number counts were calculated with Agilent’s online tool “*E. coli* Cell Culture Concentration from OD_600_ Calculator” (https://www.agilent.com/store/biocalculators/calcODBacterial.jsp, accessed 11/14/2022).

### RNA sequencing and analysis

Sampled cryo-cultures of the simulated long-term cultivation were re-cultivated for 72 h with a starting OD_600_ of 0.01 into 25 ml fresh medium under above mentioned conditions. One sample per early/late generation population, plasmid and strain (12 in total; strains: W3110, MDS42; plasmids: pCYS, pCYS_i, pCYS_m) were chosen for RNA extractions using a standard RNA extraction kit (Promega SV total RNA isolation system). Depletion of ribosomal RNA, transcriptome library construction, and sequencing of the corresponding samples were performed by Eurofins Genomics. Sequencing was conducted with the technology of Illumina NovaSeq 6000 with a 150 bp paired-end reading. Raw read counts were created using featureCounts (version 1.5.1) [55]. Only reads overlapping “CDS” features were counted. All reads mapping to features with the same meta-feature attribute were summed. Only reads with unique mapping positions and a mapping quality score of at least 10 were considered for read counting. Supplementary alignments were ignored. Instead of counting paired-end reads twice, they were counted only once, i.e. as single fragment. Reads mapping to multiple features were assigned to the feature that has the largest number of overlapping bases. A Trimmed Mean of M-values (TMM) normalization was performed using the edgeR package (version 3.16.5) [56, 57]. Mapping of reads to reference sequences of *E. coli* K-12 W3110 and *E. coli* K-12 MDS42 was performed using BWA-NEM (version 0.7.12-r1039).

To determine which features are significantly differentially expressed, a small threshold (< < 0.01) was applied to the false discovery rate (FDR) values, which is the p-value adjusted for multiple testing using Benjamini–Hochberg procedure. Fold changes were calculated by dividing values of the later generation population (LGP) by values of the early generation population (EGP).

The metabolic gene clustering was carried out with the EcoCyc *E. coli* database regarding all differentially expressed features with p-values < 0.05. Mapping results and expression profiling statistics are shown in Additional file 1: Tables S4, S5.

### Plasmid analysis of early and late generation population of *E. coli* W3110 and MDS42

Sampled cryo-cultures of the simulated long-term cultivation were re-cultivated for 72 h with a starting OD_600_ of 0.01 into 25 ml fresh medium under above mentioned conditions. One sample per early/late generation population, plasmid and strain (12 in total; strains: W3110, MDS42; plasmids: pCYS, pCYS_i, pCYS_m) were chosen for plasmid extraction, using a standard kit (ThermoFisher Scientific GeneJET Plasmid Miniprep Kit). In order to test for potential plasmid loss during cultivation, we determined the plasmid DNA content in early and late generation populations (Additional file 1: Table S12). For The subsequent library preparation and deep sequencing was performed by Eurofins Genomics for Illumina NovaSeq 6000 S4 paired-end 2 × 150 bp. The per base coverage depth was > 140.000x. Adapter trimming, quality filtering and per-read pruning was done to retain only high quality bases. Reads were then mapped to the corresponding reference plasmid sequence, followed by a single nucleotide variant calling. Reads which could not be mapped to the plasmid reference sequences, got then mapped against an insertion sequence database (ISfinder_Nucl) with the Burrows-Wheeler Aligner. The reads, which could then be mapped to insertion sequences, were blasted against each insertion sequence family in the *E. coli* W3110 genome with NCBI’s megablast algorithm. Only highly similar sequences were selected and displayed.

## Supplementary Information

**Additional file 1**: **Table S1**. Oligo sequences used for assembly of plasmids pCYS_i and pCYS_m. **Table S2**. Average number of generations after each passage of the simulated fermentation of W3110 and MDS42 with integrated pCYS, pCYS_i and pCYS_m. Each strain was cultivated in biological triplicates. **Table S3**. Average growth rates of samples taken after each passage of the simulated long-term cultivation of W3110 and MDS42 with integrated pCYS, pCYS_i and pCYS_m. Each strain was cultivated in biological triplicates. Growth rates were calculated according to the formula found in the manuscripts’ methods section of “Measurement of population growth rates”. **Table S4**. Mapping statistics overview. For each sample, the following statistics are provided: Reads mapped: the total number of reads mapped to the reference genome. Unique: number of uniquely mapped reads, i.e. read can only be mapped to one reference locus. Reference covered: reference bases covered by at least one read. Mean read coverage: average read coverage of the reference sequence. HGP: high generation population, LGP: low generation population. **Table S5**. Expression profiling statistics. For each sample, the following statistics are provided: Effective library size: The total number of reads mapped to reference features. Normalized library size: The total number of reads mapped to reference features normalized by the associated normalization factor, which can be derived by dividing the normalized library size by the effective library size. No feature: The number of reads mapping to the reference sequence that could not be assigned to any annotated feature, i.e. mapping positions and feature positions do not overlap. Filtered: The number of reads that were filtered due to insufficient mapping quality or ambiguous mapping location. These reads were ignored for read counting. **Table S6**. Table showing accession numbers, gene names, features, logarithmic fold changes (logFC) and p-values of all differentially expressed genes (DEGs) of W3310_pCYS with p-values <0.05. LogFC and logCPM were calculated by dividing values of the later generation population (LGP) by values of the early generation population (EGP). *: Genes were excluded because they did not fall within the FC range of the metabolic cluster. **Table S7**. Table showing accession numbers, gene names, features, logarithmic fold changes (logFC) and p-values of all differentially expressed genes (DEGs) of MDS42_pCYS with p-values <0.05. LogFC and logCPM were calculated by dividing values of the later generation population (LGP) by values of the early generation population (EGP). **Table S8. **Table showing accession numbers, gene names, features, logarithmic fold changes (logFC) and p-values of all differentially expressed genes (DEGs) of W3110_pCYS_i with p-values <0.05. LogFC and logCPM were calculated by dividing values of the later generation population (LGP) by values of the early generation population (EGP). **Table S9. **Table showing accession numbers, gene names, features, logarithmic fold changes (logFC) and p-values of all differentially expressed genes (DEGs) of MDS42_pCYS_i with p-values <0.05. LogFC and logCPM were calculated by dividing values of the later generation population (LGP) by values of the early generation population (EGP). **Table S10**. Table showing accession numbers, gene names, features, logarithmic fold changes (logFC) and p-values of all differentially expressed genes (DEGs) of W3110_pCYS_m with p-values <0.05. LogFC and logCPM were calculated by dividing values of the later generation population (LGP) by values of the early generation population (EGP). **Table S11**. Table showing accession numbers, gene names, features, logarithmic fold changes (logFC) and p-values of all differentially expressed genes (DEGs) of MDS42_pCYS_m with p-values <0.05. LogFC and logCPM were calculated by dividing values of the later generation population (LGP) by values of the early generation population (EGP). **Table S12**. Values of calculated plasmid DNA contents extracted from early and late generation populations (EGP, LGP). Plasmid DNA was extracted from 10 ml cultures and eluted in 50 µl each. Cell dry weights were extrapolated with the factor of 0.33 g/L/OD_600= 1.0_ for *E. coli* K-12 MG1655 cells according to Sauer et al. (2). **Table S13**. Single-nucleotide polymorphism (SNP) variant table. The SNP calling was done using VarSCan2 (3). Allele frequency cut-off used for variant calling was 1%. For each sample, the following variant summary is provided: Strain, plasmid, population. Additionally, POS: Position at which the variant was observed, BB/ORF: Location affected by the mutation (BB: backbone, ORF: open reading frame), REF: Reference base, ALT: Alternative base, Allele Freq: Variant allele frequency in percentage, Alt Depth: Depth of variant-supporting bases, total depth: Depth of variant-supporting bases and reference-supporting bases. Mutations which showed allele-frequencies >95% were assumed to be originally present in the plasmids. **Figure S1**. Plots of total L-cysteine yields in mg/OD_600_ = 1.0. Cultivation and subsequent L-cysteine yields determination was carried out in biological triplicates of W3110 (A) and MDS42 (B) with the three different plasmids pCYS, pCYS_i and pCYS_m. **Figure S2**. Per base coverage depths (x) of sequenced pCYS extracted from early and late generation populations (EGPs and LGPs) of *E. coli* W3110 and MDS42. Sequencing was conducted with Illumina Novaseq paired end 2x150bp. **Figure S3. **Per base coverage depths (x) of sequenced pCYS_i extracted from early and late generation populations (EGPs and LGPs) of *E. coli* W3110 and MDS42. Sequencing was conducted with Illumina Novaseq paired end 2x150bp. **Figure S4**. Per base coverage depths (x) of sequenced pCYS_m extracted from early and late generation populations (EGPs and LGPs) of *E. coli* W3110 and MDS42. Sequencing was conducted with Illumina Novaseq paired end 2x150bp. **Note 1**. Spectrophotometric protocol for L-Cysteine determination adapted from Gaitonde (1).

## Data Availability

All data and materials are available as described in the study and its additional Additional file 1.

## Abbreviations

ALE: Adaptive laboratory evolution
*C. Glutamicum*: *Corynebacterium glutamicum*
DEG: Differentially expressed gene
*E. coli*: *Escherichia coli*
EGP: Early generation population
IS: Insertion sequence
LGP: Late generation population
NGS: Next generation sequencing
R.P.M: Rounds per minute
SNP: Single-nucleotide polymorphism
