# Supplementary material for: Metabolic stress constrains microbial L-cysteine production in Escherichia coli by accelerating transposition through mobile genetic elements
Source: Microb Cell Fact. 2023 Jan 16;22:10. doi: 10.1186/s12934-023-02021-5 (PMC9841684; doi:10.1186/s12934-023-02021-5)
Supplement: Supplementary file 1 — Additional file 1: Table S1. Oligo sequences used for assembly of plasmids pCYS_i and pCYS_m. Table S2. Average number of generations after each passage of the simulated fermentation of W3110 and MDS42 with integrated pCYS, pCYS_i and pCYS_m. Each strain was cultivated in biological triplicates. Table S3. Average growth rates of samples taken after each passage of the simulated long-term cultivation of W3110 and MDS42 with integrated pCYS, pCYS_i and pCYS_m. Each strain was cultivated in biological triplicates. Growth rates were calculated according to the formula found in the manuscripts’ methods section of “Measurement of population growth rates”. Table S4. Mapping statistics overview. For each sample, the following statistics are provided: Reads mapped: the total number of reads mapped to the reference genome. Unique: number of uniquely mapped reads, i.e. read can only be mapped to one reference locus. Reference covered: reference bases covered by at least one read. Mean read coverage: average read coverage of the reference sequence. HGP: high generation population, LGP: low generation population. Table S5. Expression profiling statistics. For each sample, the following statistics are provided: Effective library size: The total number of reads mapped to reference features. Normalized library size: The total number of reads mapped to reference features normalized by the associated normalization factor, which can be derived by dividing the normalized library size by the effective library size. No feature: The number of reads mapping to the reference sequence that could not be assigned to any annotated feature, i.e. mapping positions and feature positions do not overlap. Filtered: The number of reads that were filtered due to insufficient mapping quality or ambiguous mapping location. These reads were ignored for read counting. Table S6. Table showing accession numbers, gene names, features, logarithmic fold changes (logFC) and p-values of all differentially express [file 12934_2023_2021_MOESM1_ESM.docx]

**Additional file**

**Heieck et al.,**

**Metabolic stress constrains microbial L-cysteine production in *Escherichia coli* by accelerating transposition through mobile genetic elements**

**Additional file 1: Table S1.** Oligo sequences used for assembly of plasmids pCYS_i and pCYS_m

| **Primer name** | **DNA sequence (5’->3’)** | **Associated assembled plasmid** |
| --- | --- | --- |
| Fragment 1_fw | CCGGAGCTCCCGCTTGACGCTGCGTAAGGTTTTTGTAATTTTACAGGCTACCTAGCACTTCGGTTTTATTTTAGGAGAACTTTAATGTCGTGTGAAGAACTGGAA | pCYS_i |
| Fragment 1_rev | TCATCACCTCGAGTTACGTATTAATCCATTGATGGCTTTCGCTGTCTGG |  |
| Fragment 2_fw | ATACTCGAGGGAAAAAGATGAAATTCAGAGGCG |  |
| Fragment 2_rev | AATGGATCCGGCTTATTAACTTCCCACC |  |
| Fragment 3_fw | TGGGGATCCGCTTATGTTAAGTACAGTCACACTACATGCAAATGATCAAAGGC |  |
| Fragment 3_rev | GGAGCCTTAATTAAGGCGTCAGATCATTTCACAATGGT |  |
| Fragment 4_fw | AGTAGTTTAATTAAGAGTCCTGGCTAACCCACAAGAAGGTTTCAAATGGCAAAGGTATCGCTG |  |
| Fragment 4_rev | ATATCGCCATGGCTGGAGTACTTAGTCAGAATACTT |  |
| Pfic_fw | CCTGAGGCTGCAGCTGCCGTAATGATTT | pCYS_m |
| Pfic_rev | TTCTAATGTACTCATATGTTGATGCCTCCCTGAACGT |  |
| cysM_fw | CAACATATGAGTACATTAGAACAAACAATAGGCAATACGCCTCTGGTGA |  |
| cysM_rev | GGGAGAGCCTGAGCAAACTGGCCTCAGGTTTAAAAGATAAAAAACGCCCGGCGGCAACCGAGCGTTCTTAAGCCGC |  |

**Additional file 1: Table S2.** Average number of generations after each passage of the simulated fermentation of W3110 and MDS42 with integrated pCYS, pCYS_i and pCYS_m. Each strain was cultivated in biological triplicates.

| **W3110** | | | | | | |
| --- | --- | --- | --- | --- | --- | --- |
|  | **pCYS** | | **pCYS_i** | | **pCYS_m** | |
| Time [h] | Accumulated generations | STD | Accumulated generations | STD | Accumulated generations | STD |
| Preculture (0) | 2.67 | 0.08 | 4.50 | 0.14 | 3.83 | 0.07 |
| 10 | 9.52 | 0.14 | 9.53 | 0.05 | 9.57 | 0.003 |
| 20 | 14.37 | 0.21 | 14.84 | 0.13 | 14.61 | 0.05 |
| 30 | 20.16 | 0.21 | 20.13 | 0.24 | 19.65 | 0.05 |
| 40 | 26.13 | 0.25 | 25.33 | 0.32 | 24.63 | 0.03 |
| 50 | 32.23 | 0.31 | 30.66 | 0.43 | 29.68 | 0.05 |
| 60 | 38.53 | 0.33 | 35.79 | 0.47 | 34.65 | 0.02 |
| 70 | 45.12 | 0.35 | 41.11 | 0.58 | 39.77 | 0.04 |
| 80 | 51.71 | 0.37 | 46.42 | 0.70 | 45.13 | 0.21 |
| 90 | 58.25 | 0.43 | 51.82 | 0.77 | 51.07 | 0.62 |
| 100 | 64.69 | 0.42 | 57.40 | 0.90 | 57.48 | 0.65 |
| 110 | - | - | 63.52 | 0.97 | 64.10 | 0.62 |
| **MDS42** | | | | | | |
| Preculture (0) | 2.70 | 0.30 | 5.21 | 0.08 | 5.64 | 0.14 |
| 5 | 6.85 | 0.42 | 8.75 | 0 | 9.26 | 0.01 |
| 10 | 11.05 | 0.42 | 12.88 | 0.04 | 13.29 | 0.15 |
| 15 | 14.93 | 0.86 | 17.09 | 0.07 | 16.84 | 0.41 |
| 20 | 18.74 | 1.22 | 20.75 | 0.09 | 21.19 | 0.32 |
| 25 | 22.60 | 1.62 | 25.04 | 0.12 | 25.24 | 0.39 |
| 30 | 26.40 | 1.98 | 29.26 | 0.11 | 29.41 | 0.39 |
| 35 | 30.35 | 2.33 | 33.37 | 0.10 | 33.54 | 0.37 |
| 40 | 34.32 | 2.64 | 37.48 | 0.11 | 37.27 | 0.34 |
| 45 | 38.29 | 2.93 | 41.73 | 0.08 | 41.29 | 0.42 |
| 50 | 42.66 | 3.15 | 46.14 | 0.14 | 45.45 | 0.41 |
| 55 | 47.05 | 3.28 | 50.34 | 0.16 | 49.59 | 0.43 |
| 60 | 51.39 | 3.36 | 54.35 | 0.26 | 53.42 | 0.40 |
| 65 | 55.63 | 3.38 | 58.56 | 0.20 | 57.83 | 0.31 |
| 70 | 60.63 | 3.37 | 62.81 | 0.19 | 62.31 | 0.21 |

**Additional file 1: Table S3.** Average growth rates of samples taken after each passage of the simulated long-term cultivation of W3110 and MDS42 with integrated pCYS, pCYS_i and pCYS_m. Each strain was cultivated in biological triplicates. Growth rates were calculated according to the formula found in the manuscripts’ methods section of “Measurement of population growth rates”.

| **W3110** | | | | | | | | |
| --- | --- | --- | --- | --- | --- | --- | --- | --- |
|  | **pCYS** | | **pCYS_i** | | **pCYS_m** | | **Empty vector** | |
| Generation [#] | Growth rate µ | STD | Growth rate µ | STD | Growth rate µ | STD | Growth rate µ | STD |
| 10 | 0.42 | 0.006 | 0.51 | 0.009 | 0.48 | 0.004 | 0.79 | 0.023 |
| 15 | - | - | 0.51 | 0.014 | 0.48 | 0.004 | - | - |
| 20 | 0.45 | 0.003 | - | - | - | - | 0.77 | 0.045 |
| 25 | - | - | - | - | 0.49 | 0.010 | - | - |
| 30 | - | - | 0.52 | 0.007 | - | - | 0.79 | 0.060 |
| 32 | 0.45 | 0.012 | - | - | - | - | - | - |
| 40 | - | - | - | - | 0.49 | 0.010 | 0.81 | 0.041 |
| 45 | 0.54 | 0.012 | - | - | - | - | - | - |
| 51 | - | - | 0.5 | 0.013 | 0.54 | 0.028 | 0.80 | 0.063 |
| 52 | 0.54 | 0.025 | - | - | - | - | - | - |
| 59 | 0.70 | 0.067 | 0.49 | 0.033 | - | - | - | - |
| 63 | 0.63 | 0.007 | 0.54 | 0.029 | 0.55 | 0.006 | 0.72 | 0.030 |
| **MDS42** | | | | | | | | |
| 7 | 0.60 | 0.030 | - | - | - | - | - | - |
| 9 | - | - | 0.78 | 0.018 | 0.79 | 0.023 | 0.68 | 0.045 |
| 14 | 0.58 | 0.010 | - | - | - | - | - | - |
| 17 | - | - | 0.80 | 0.013 | - | - | 0.67 | 0.030 |
| 25 | - | - | 0.81 | 0.008 | 0.79 | 0.017 | - | - |
| 26 | 0.58 | 0.020 | - | - | - | - | 0.67 | 0.051 |
| 32 | - | - | - | - | 0.80 | 0.015 | - | - |
| 45 | - | - | 0.81 | 0.088 | 0.79 | 0.017 | 0.69 | 0.042 |
| 47 | 0.58 | 0.008 | - | - | - | - | - | - |
| 60 | 0.57 | 0.041 | - | - | - | - | 0.66 | 0.063 |
| 62 | - | - | 0.81 | 0.004 | 0.79 | 0.015 | - | - |

**Additional file Note 1**: Spectrophotometric protocol for L-Cysteine determination adapted from Gaitonde (1).

While the supernatant was transferred directly to a new 1.5 ml reaction tube, the pellet was incubated with 1 ml of mixture 1 (composition below) and shaken for 2 h in a Thermomix (Eppendorf) with 1.000 rpm at 70°C. Samples were then centrifuged at 14,000 x g for 5 min and the supernatant transferred to a new 1.5 ml reaction tube. 100 µl of both reaction mixtures (supernatant and pellet) were incubated with 385 µl of Tris-HCl buffer (pH 8) and 30 µl of a freshly prepared 0.1 M solution of dithiothreitol (DTT) in 2.0 ml reaction tubes. This mixture was incubated for 10 min at room temperature to reduce potential disulphide bonds. Then, 500 µl of ninhydrin reagent (composition below) were added to the mixture and heated at 100°C for 10 min. In order to stabilize the pink product, 1 ml of 95% EtOH was added and cooled on ice. Finally, the absorbance was measured at 560 nm. A sample with minimal medium subjected to the same procedure was used as a blank. Additionally, ninhydrin assays of W3110 and MDS42 cultured in minimal medium with transformed empty vectors were performed. These negative controls showed no absorption at all. A calibration curve of the A560 absorbance as a function of different L-cysteine concentrations was performed with L-cysteine (98.5%) (Sigma) (see below).

**Mixture 1 (Storage at room temperature)**

| **Compound** | **Volume [ml]** |
| --- | --- |
| Phosphoric acid [85%] | 15.25 |
| Sulphuric acid [96%] | 1.53 |
| H_2_O_dd._ | 83.22 |

**Ninhydrin reagent (Storage at 4°C for up to 2 weeks)**

| **Compound** | **Amount** |
| --- | --- |
| Ninhydrin | 1.25 g |
| Hydrochloride acid | 20 ml |
| Acetic acid | 80 ml |

Calibration curve of the A560 absorbance as a function of different L-cysteine concentrations performed with L-cysteine (98.5%) from Sigma. Concentrations from 0.1 g/L to 0.9 g/L were measured. The function y = 0.9335x + 0.2116 of the according trend line was used to calculate L-cysteine concentrations.


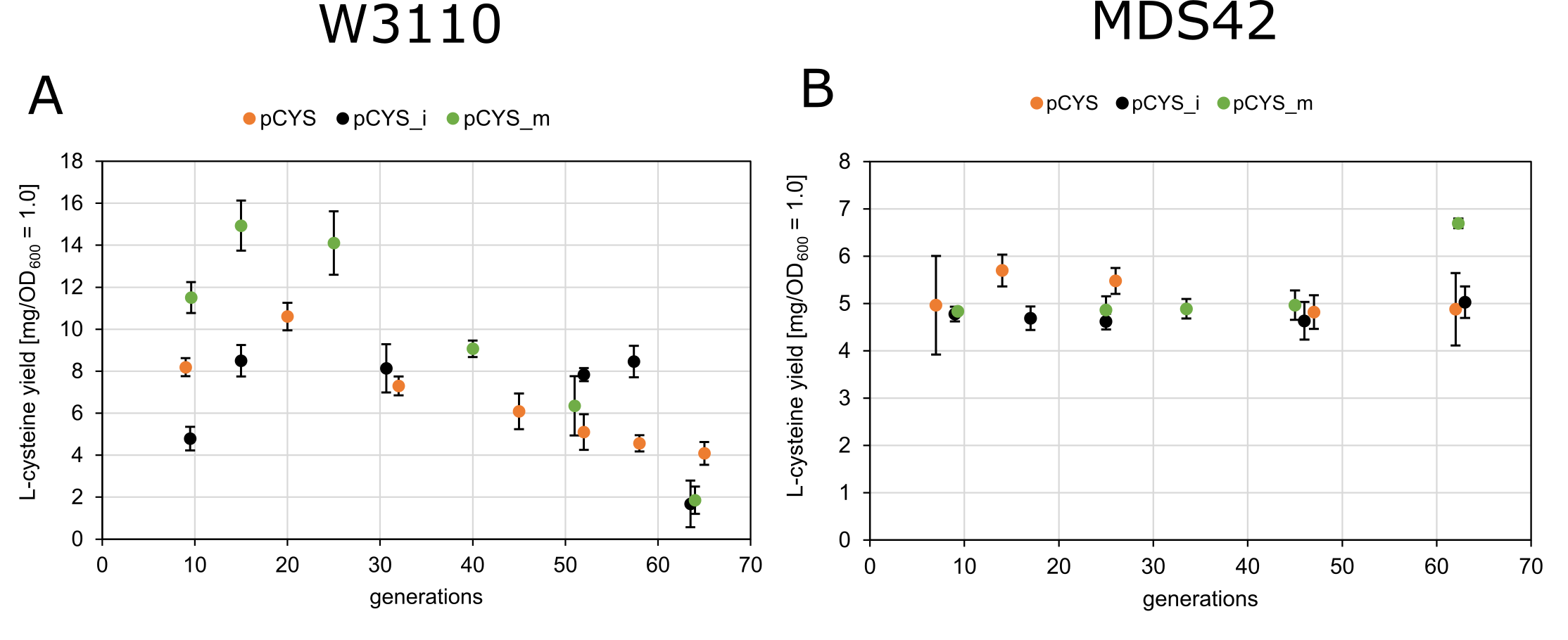


**Additional file 1: figure S1:** Plots of total L-cysteine yields in mg/OD_600_ = 1.0. Cultivation and subsequent L-cysteine yields determination was carried out in biological triplicates of W3110 (A) and MDS42 (B) with the three different plasmids pCYS, pCYS_i and pCYS_m.

**Additional file 1: table S4:** Mapping statistics overview. For each sample, the following statistics are provided: Reads mapped: the total number of reads mapped to the reference genome. Unique: number of uniquely mapped reads, i.e. read can only be mapped to one reference locus. Reference covered: reference bases covered by at least one read. Mean read coverage: average read coverage of the reference sequence. HGP: high generation population, LGP: low generation population.

| **Sample** | **Reads mapped [Mio]** | **Unique [Mio]** | **Reference covered [Mb]** |
| --- | --- | --- | --- |
| W3110_pCYS_HGP | 15.74 (98.6%) | 15.43 (96.6%) | 4.35 (93.7%) |
| W3110_pCYS_LGP | 17.48 (97.5%) | 17.16 (95.7%) | 4.26 (91.8%) |
| MDS42_pCYS_HGP | 12.52 (98.2%) | 12.35 (96.9%) | 3.54 (76.3%) |
| MDS42_pCYS_LGP | 21.15 (98.1%) | 20.73 (96.1%) | 2.63 (78.2%) |
| W3110_pCYS_i_HGP | 63.00 (98.0%) | 62.31 (96.9%) | 4.61 (99.2%) |
| W3110_pCYS_i_LGP | 38.42 (97.1%) | 37.97 (96.0%) | 4.56 (98.3%) |
| MDS42_pCYS_i_HGP | 52.81 (98.3%) | 52.30 (97.3%) | 3.93 (84.6%) |
| MDS42_pCYS_i_LGP | 32.11 (98.5%) | 31.88 (97.8%) | 3.89 (83.7%) |
| W3110_pCYS_m_HGP | 13.74 (98.5%) | 13.62 (97.7%) | 4.59 (99.1%) |
| W3110_pCYS_m_LGP | 10.93 (97.3%) | 10.84 (96.5%) | 4.35 (97.4%) |
| MDS42_pCYS_m_HGP | 12.75 (98.4%) | 12.64 (97.6%) | 3.89 (84%) |
| MDS42_pCYS_m_LGP | 13.14 (97.9%) | 13.05 (97.3%) | 3.76 (83.3%) |

**Additional file 1: table S5:** Expression profiling statistics. For each sample, the following statistics are provided: Effective library size: The total number of reads mapped to reference features. Normalized library size: The total number of reads mapped to reference features normalized by the associated normalization factor, which can be derived by dividing the normalized library size by the effective library size. No feature: The number of reads mapping to the reference sequence that could not be assigned to any annotated feature, i.e. mapping positions and feature positions do not overlap. Filtered: The number of reads that were filtered due to insufficient mapping quality or ambiguous mapping location. These reads were ignored for read counting.

| **Sample** | **Effective Library Size** | **Normalized Library Size** | **No Feature** | **Filtered** |
| --- | --- | --- | --- | --- |
| W3110_pCYS_HGP | 14,252,994 | 14,476,778.3 | 1,165,261 | 538,202 |
| W3110_pCYS_LGP | 15,767,859 | 17,145,605.9 | 1,379,155 | 776,008 |
| MDS42_pCYS_HGP | 10,911,912 | 11,159,581.3 | 1,433,356 | 390,282 |
| MDS42_pCYS_LGP | 18,559,438 | 17,419,716.3 | 2,160,469 | 832,562 |
| W3110_pCYS_i_HGP | 28,130,536 | 27,532,366.2 | 2,882,373 | 1,119,315 |
| W3110_pCYS_i_LGP | 16,502,506 | 18,445,755.2 | 2,402,562 | 869,322 |
| MDS42_pCYS_i_HGP | 23,361,202 | 21,780,445.7 | 2,574,658 | 915,004 |
| MDS42_pCYS_i_LGP | 14,731,777 | 13,971,444.8 | 981,102 | 571,985 |
| W3110_pCYS_m_HGP | 18,597,401 | 18,489,582 | 1,065,231 | 481,982 |
| W3110_pCYS_m_LGP | 18,351,569 | 18,154,799 | 995,471 | 571,451 |
| MDS42_pCYS_m_HGP | 17,581,965 | 17,281,118 | 1,921,667 | 581,949 |
| MDS42_pCYS_m_LGP | 16,411,682 | 15,984,381 | 1,763,955 | 739,814 |

**Additional file 1: table S6:** Table showing accession numbers, gene names, features, logarithmic fold changes (logFC) and p-values of all differentially expressed genes (DEGs) of W3310_pCYS with p-values <0.05. LogFC and logCPM were calculated by dividing values of the later generation population (LGP) by values of the early generation population (EGP). *: Genes were excluded because they did not fall within the FC range of the metabolic cluster.

| **Accession** | **Gene** | **Feature** | **LogFC** | **P-Value** |
| --- | --- | --- | --- | --- |
| b4205 | *ytfA* | biofilm formation | 6.31 | 0.0004 |
| b3110 | *cyuP* | l-cysteine degradation | 5.12 | 0.001 |
| b1505 | *ydeT* | biofilm formation | 5.07 | 0.001 |
| b1556 | *essQ* | unknown function | 5.12 | 0.002 |
| b1504 | *YdeS* | biofilm formation | 5.02 | 0.003 |
| b4293 | *FecI* | iron starvation | 4.28 | 0.003 |
| b1503 | *ydeR* | biofilm formation | 4.95 | 0.003 |
| b4002 | *ZraP* | biofilm formation | 4.25 | 0.004 |
| b1560 | *YdfU* | unknown function | 4.23 | 0.004 |
| b4470 | *CyuA* | l-cysteine degradation | 4.11 | 0.004 |
| b4277 | *yjgZ* | insertion sequence element | 5.05 | 0.004 |
| b1038 | *csgF* | curli secretion | -4.15 | 0.004 |
| b3558 | *insK* | insertion sequence element | 4.05 | 0.005 |
| b0585 | *fes* | iron starvation | 4.04 | 0.005 |
| b4292 | *fecR* | iron starvation | 4.00 | 0.005 |
| b0584 | *fepA* | iron starvation | 3.98 | 0.005 |
| b0590 | *fepD* | iron starvation | 3.99 | 0.005 |
| b4567 | *yjjZ* | unknown function | 4.20 | 0.005 |
| b0691 | *ybfG* | biofilm formation | 7.66 | 0.006 |
| b1309 | *ycjM* | alternative sugar utilizing pathway | 4.36 | 0.007 |
| b4011 | *yjaA* | biofilm formation | 3.87 | 0.007 |
| b1502 | *ydeQ* | biofilm formation | 4.05 | 0.007 |
| b2229 | *yfaT* | unknown function | 4.40 | 0.007 |
| b0587 | *fepE* | iron starvation | 7.53 | 0.007 |
| b0236 | *prfH* | unknown function | 4.30 | 0.008 |
| b2298 | *yfcC* | unknown function | 3.65 | 0.010 |
| b0589 | *fepG* | iron starvation | 3.61 | 0.010 |
| b1554 | *rrrQ* | unknown function | 3.73 | 0.011 |
| b0986 | *gfC* | unknown function | 3.72 | 0.011 |
| b1039 | *csgE* | curli secretion | -3.53 | 0.012 |
| b1600 | *mdtJ* | multidrug efflux | 3.55 | 0.013 |
| b0583 | *entD* | iron starvation | 3.46 | 0.013 |
| b2221 | *atoD* | acetyl-coA metabolic process | -3.56 | 0.014 |
| b3557 | *insJ* | Insertion sequence element | 3.34 | 0.016 |
| b1553 | *rzpQ* | unknown function | 3.55 | 0.017 |
| b1496 | *yddA* | iron starvation | 3.81 | 0.017 |
| b1037 | *csgG* | curli secretion | -3.25 | 0.018 |
| b1599 | *mdtl* | multidrug efflux | 3.32 | 0.019 |
| b1466 | *narW* | nitrate assimilation | -3.27 | 0.019 |
| b3800 | *aslB* | L-cysteine degradation | 3.20 | 0.020 |
| b0985 | *gfcC* | biofilm formation | 3.29 | 0.020 |
| b2155 | *cirA* | iron starvation | 3.18 | 0.020 |
| b0591 | *entS* | iron starvation | 3.15 | 0.022 |
| b3158 | *ubiU* | ubiquinone biosynthesis | 3.15 | 0.022 |
| b0894 | *dmsA* | anaerobic respiration | 3.07 | 0.024 |
| b0549 | *ybcO* | unknown function | 3.67 | 0.024 |
| b1492 | *gadC* | acid resistance | -3.05 | 0.025 |
| b3708 | *tnaA* | tryptophanases | -3.06 | 0.025 |
| b3060 | *ttdR* | transcriptional regulator | 3.15 | 0.025 |
| b0575 | *cusA* | copper/silver export | 3.04 | 0.025 |
| b2343 | *yfcZ* | unknown function | 3.02 | 0.026 |
| b1493 | *gadB* | acid resistance | -3.00 | 0.028 |
| b1040 | *csgD* | curli secretion | -2.98 | 0.028 |
| b4183 | *yjkK* | unknown function | 3.21 | 0.029 |
| b2339 | *yfcV* | biofilm formation | 3.48 | 0.029 |
| b2997 | *hybO* | anaerobic respiration | 2.94 | 0.030 |
| b2534 | *yfhR* | unknown function | 3.12 | 0.032 |
| b0547 | *ybcN* | mismatch repair | 3.17 | 0.032 |
| b0593 | *entC* | iron starvation | 2.90 | 0.032 |
| b0984 | *gfcD* | unknown function | 2.92 | 0.033 |
| b3408 | *feoA* | iron starvation | 2.89 | 0.033 |
| b4511 | *ybdZ* | iron starvation | 3.07 | 0.033 |
| b2222 | *atoA* | acetyl-coA metabolic process | -3.00 | 0.034 |
| b1409 | *ynbB* | biofilm formation | 3.10 | 0.035 |
| b1674 | *ydhY* | iron starvation | 3.02 | 0.036 |
| b1468 | *narZ* | nitrate assimilation | -2.81 | 0.037 |
| b3654 | *xanP* | xanthine transport | 2.81 | 0.037 |
| b0913 | *ycal* | unknown function | 2.80 | 0.038 |
| b0836 | *bssR* | biofilm formation | 2.79 | 0.038 |
| b1469 | *narU* | nitrate assimilation | -2.79 | 0.039 |
| b2172 | *yeiQ* | unknown function | 2.78 | 0.039 |
| b3020 | *ygiS* | unknown function | 2.77 | 0.039 |
| b1121 | *ycfZ* | unknown function | 4.17 | 0.040 |
| b0550 | *rusA* | mismatch repair | 2.98 | 0.042 |
| b1161 | *ycgX* | unknown function | 3.42 | 0.043 |
| b4367 | *fhuF* | iron starvation | 2.72 | 0.043 |
| b0375 | *iprA* | mismatch repair | 4.08 | 0.046 |
| b0574 | *cusB* | copper/silver export | 2.68 | 0.046 |
| b1541 | *ydfZ* | unknown function | 2.71 | 0.046 |
| b1467 | *narY* | nitrate reductase | -2.67 | 0.047 |
| b4314 | *fimA** | biofilm formation | -2.63 | 0.049 |

**Additional file 1: table S7:** Table showing accession numbers, gene names, features, logarithmic fold changes (logFC) and p-values of all differentially expressed genes (DEGs) of MDS42_pCYS with p-values <0.05. LogFC and logCPM were calculated by dividing values of the later generation population (LGP) by values of the early generation population (EGP).

| **Accession** | **Gene** | **Feature** | **LogFC** | **P-Value** |
| --- | --- | --- | --- | --- |
| b4035 | *malK* | maltose transport | -4.54 | 0.0005 |
| b4036 | *lamB* | maltose transport | -4.03 | 0.0013 |
| b1224 | *narG* | nitrate assimilation | 3.86 | 0.0019 |
| b1225 | *narH* | nitrate assimilation | 3.78 | 0.0022 |
| b1223 | *narK* | nitrate assimilation | 3.74 | 0.0025 |
| b3060 | *ttdR* | transcriptional regulator | 3.56 | 0.0043 |
| b1226 | *narJ* | nitrate assimilation | 3.40 | 0.0052 |
| b4034 | *malE* | maltose transport | -3.34 | 0.0064 |
| b1227 | *narI* | nitrate assimilation | 3.30 | 0.0065 |
| b4037 | *malM* | maltose transport | -3.00 | 0.0127 |
| b4242 | *mgtA* | Mg2+ transport | -2.96 | 0.0131 |
| b4032 | *malG* | maltose transport | -3.01 | 0.0133 |
| b1750 | *zdjX* | unknown function | 3.00 | 0.0145 |
| b1436 | *yncJ* | unknown function | -3.87 | 0.0193 |
| b0894 | *dmsA* | anaerobic respiration | 2.65 | 0.0243 |
| b4702 | *mgtL* | Mg2+ transport | -3.13 | 0.0254 |
| b4033 | *malF* | maltose transport | -2.70 | 0.0259 |
| b3366 | *nirD* | nitrate assimilation | 2.59 | 0.0274 |
| b2111 | *yehD* | biofilm formation | 3.12 | 0.0279 |
| b2298 | *yfcC* | unknown function | 2.56 | 0.0303 |
| b2243 | *glpC* | anaerobic respiration | 2.53 | 0.0307 |
| b3367 | *nirC* | nitrate assimilation | 2.50 | 0.0329 |
| b0836 | *bssR* | biofilm formation | 2.44 | 0.0368 |
| b3158 | *ubiU* | ubiquinone biosynthesis | 2.45 | 0.0375 |
| b3960 | *argH* | L-arginine biosynthesis | 2.43 | 0.0375 |
| b1826 | *mgrB* | Mg2+ transport | -2.51 | 0.0395 |
| b1748 | *astC* | L-arginine degradation | -2.37 | 0.0426 |
| b3365 | *nirB* | nitrate assimilation | 2.36 | 0.0429 |
| b2242 | *glpB* | anaerobic respiration | 2.36 | 0.0430 |
| b1182 | *hlyE* | hemolysin | 2.67 | 0.0435 |
| b0895 | *dmsB* | anaerobic respiration | 2.35 | 0.0438 |
| b3508 | *yhiD* | unknown function | 2.52 | 0.0448 |
| b1608 | *rstA* | unknown function | -2.34 | 0.0451 |
| b1751 | *ydjy* | unknown function | 2.30 | 0.0498 |

**Additional file 1: table S8:** Table showing accession numbers, gene names, features, logarithmic fold changes (logFC) and p-values of all differentially expressed genes (DEGs) of W3110_pCYS_i with p-values <0.05. LogFC and logCPM were calculated by dividing values of the later generation population (LGP) by values of the early generation population (EGP).

| **Accession** | **Gene** | **Feature** | **LogFC** | **P-Value** |
| --- | --- | --- | --- | --- |
| b0365 | *tauA* | sulfur/L-cysteine starvation | -10.82 | 4.37E-07 |
| b0366 | *tauB* | sulfur/L-cysteine starvation | -10.57 | 6.44E-07 |
| b0367 | *tauC* | sulfur/L-cysteine starvation | -9.97 | 1.60E-06 |
| b0368 | *tauD* | sulfur/L-cysteine starvation | -8.97 | 7.24E-06 |
| b0937 | *ssuE* | sulfur/L-cysteine starvation | -8.61 | 2.22E-05 |
| b3917 | *sbP* | sulfur/L-cysteine starvation | -7.98 | 3.27E-05 |
| b0935 | *ssUD* | sulfur/L-cysteine starvation | -7.91 | 3.94E-05 |
| b0936 | *ssuA* | sulfur/L-cysteine starvation | -7.36 | 9.94E-05 |
| b0934 | *ssuC* | sulfur/L-cysteine starvation | -6.63 | 2.81E-04 |
| b2752 | *cysD* | sulfur/L-cysteine starvation | -6.56 | 2.92E-04 |
| b2750 | *cysC* | sulfur/L-cysteine starvation | -6.54 | 3.01E-04 |
| b2751 | *cysN* | sulfur/L-cysteine starvation | -6.24 | 4.68E-04 |
| b2422 | *cysA* | sulfur/L-cysteine starvation | -5.51 | 1.43E-03 |
| b0933 | *ssUB* | sulfur/L-cysteine starvation | -5.37 | 1.81E-03 |
| b2763 | *cysI* | sulfur/L-cysteine starvation | -5.16 | 2.40E-03 |
| b2424 | *cysU* | sulfur/L-cysteine starvation | -5.16 | 2.47E-03 |
| b2423 | *cysW* | sulfur/L-cysteine starvation | -5.07 | 2.80E-03 |
| b4721 | *ytiD* | unknown function | -5.99 | 3.16E-03 |
| b2762 | *cysH* | sulfur/L-cysteine starvation | -4.95 | 3.29E-03 |
| b2764 | *cysJ* | sulfur/L-cysteine starvation | -4.70 | 4.77E-03 |
| b1492 | *gadC* | acid resistance | -4.42 | 7.18E-03 |
| b1493 | *gadB* | acid resistance | -4.15 | 1.07E-02 |
| b4518 | *ymdF* | unknown function | -4.09 | 1.17E-02 |
| b3517 | *gadA* | acid resistance | -4.01 | 1.29E-02 |
| b2425 | *cysP* | sulfur/L-cysteine starvation | -3.98 | 1.38E-02 |
| b1038 | *csgF* | curli secretion | -3.98 | 1.38E-02 |
| b1039 | *csgE* | curli secretion | -3.87 | 1.61E-02 |
| b1467 | *narY* | nitrate assimilation | -3.77 | 1.84E-02 |
| b0897 | *ysaC* | unknown function | -3.73 | 1.94E-02 |
| b3477 | *nikB* | nickel transport | 3.75 | 1.97E-02 |
| b1489 | *dosP* | oxygen-sensing | -3.72 | 1.98E-02 |
| b3491 | *yhiM* | unknown function | -3.72 | 1.99E-02 |
| b2379 | *alaC* | L-alanine biosynthesis | -3.71 | 2.00E-02 |
| b3555 | *yiaG* | unknown function | -3.69 | 2.04E-02 |
| b0753 | *ybgS* | unknown function | -3.64 | 2.19E-02 |
| b0775 | *bioB* | biotin biosynthesis | 3.67 | 2.23E-02 |
| b1466 | *narW* | nitrate assimilation | -3.63 | 2.32E-02 |
| b2414 | *cysK* | L-cysteine /precursor biosynthesis | -3.58 | 2.37E-02 |
| b1287 | *yciW* | hyperosmotic stress | -3.58 | 2.40E-02 |
| b3073 | *patA* | nitrogen limitation indicator | -3.56 | 2.45E-02 |
| b1465 | *narV* | nitrate assimilation | -3.57 | 2.48E-02 |
| b1040 | *csgD* | curli secretion | -3.55 | 2.51E-02 |
| b2427 | *murR* | muramic acid regulator | -3.56 | 2.51E-02 |
| b4187 | *aidB* | cellular response to DNA damage | -3.51 | 2.64E-02 |
| b1468 | *narZ* | nitrate assimilation | -3.48 | 2.74E-02 |
| b1469 | *narU* | nitrate assimilation | -3.45 | 2.90E-02 |
| b2241 | *glpA* | anaerobic respiration | 3.43 | 3.01E-02 |
| b1259 | *yciG* | acid resistance | -3.44 | 3.15E-02 |
| b3510 | *hdeA* | acid resistance | -3.36 | 3.24E-02 |
| b0553 | *nmpC* | unknown function | 3.31 | 3.53E-02 |
| b2749 | *ygbE* | unknown function | -3.29 | 3.60E-02 |
| b3511 | *hdeD* | acid resistance | -3.25 | 3.77E-02 |
| b3478 | *nikZ* | nickel transport | 3.23 | 4.01E-02 |
| b0774 | *bioA* | biotin biosynthesis | 3.21 | 4.18E-02 |
| b2013 | *tsuA* | sulfur/L-cysteine starvation | -3.17 | 4.21E-02 |
| b1732 | *katE* | hyperosmotic stress | -3.16 | 4.25E-02 |
| b2012 | *ysuB* | sulfur/L-cysteine starvation | -3.16 | 4.26E-02 |
| b0776 | *bioF* | biotin biosynthesis | 3.16 | 4.44E-02 |
| b4376 | *osmY* | hyperosmotic stress | -3.12 | 4.49E-02 |
| b1137 | *ymfD* | unknown function | 3.40 | 4.52E-02 |
| b2465 | *tktB* | L-cysteine /precursor biosynthesis | -3.11 | 4.53E-02 |
| b4568 | *ytjA* | unknown function | -3.10 | 4.58E-02 |
| b1037 | *csgG* | curli secretion | -3.10 | 4.60E-02 |
| b3514 | *mdtF* | multidrug efflux | -3.08 | 4.75E-02 |
| b3661 | *nlpA* | methionine transport | -3.05 | 4.89E-02 |

**Additional file 1: table S9:** Table showing accession numbers, gene names, features, logarithmic fold changes (logFC) and p-values of all differentially expressed genes (DEGs) of MDS42_pCYS_i with p-values <0.05. LogFC and logCPM were calculated by dividing values of the later generation population (LGP) by values of the early generation population (EGP).

| **Accession** | **Gene** | **Feature** | **LogFC** | **P-Value** |
| --- | --- | --- | --- | --- |
| b0936 | *ssuA* | sulfur-/L-Cysteine starvation | -4.48 | 0.0068 |
| b0937 | *ssuE* | sulfur-/L-Cysteine starvation | -4.43 | 0.0075 |
| b3603 | *lldP* | cellular response to DNA damage | 4.35 | 0.0081 |
| b3917 | *sbp* | sulfur-/L-Cysteine starvation | -4.33 | 0.0082 |
| b0934 | *ssuC* | sulfur-/L-Cysteine starvation | -4.20 | 0.0101 |
| b0935 | *ssuD* | sulfur-/L-Cysteine starvation | -4.06 | 0.0122 |
| b2752 | *cysD* | sulfur-/L-Cysteine starvation | -4.03 | 0.0128 |
| b3604 | *lldR* | cellular response to DNA damage | 4.03 | 0.0130 |
| b3605 | *lldD* | cellular response to DNA damage | 3.99 | 0.0134 |
| b2751 | *cysN* | sulfur-/L-Cysteine starvation | -3.78 | 0.0180 |
| b2764 | *cysJ* | sulfur-/L-Cysteine starvation | -3.77 | 0.0184 |
| b2763 | *cysI* | sulfur-/L-Cysteine starvation | -3.64 | 0.0219 |
| b2750 | *cysC* | sulfur-/L-Cysteine starvation | -3.62 | 0.0226 |
| b2425 | *cysP* | sulfur-/L-Cysteine starvation | -3.56 | 0.0250 |
| b2013 | *tsuA* | sulfur-/L-Cysteine starvation | -3.55 | 0.0253 |
| b2012 | *tsuB* | sulfur-/L-Cysteine starvation | -3.52 | 0.0264 |
| b2422 | *cysA* | sulfur-/L-Cysteine starvation | -3.43 | 0.0294 |
| b2762 | *cysH* | sulfur-/L-Cysteine starvation | -3.42 | 0.0300 |
| b0933 | *ssuB* | sulfur-/L-Cysteine starvation | -3.37 | 0.0321 |
| b2424 | *cysU* | sulfur-/L-Cysteine starvation | -3.27 | 0.0372 |
| b2423 | *cysW* | sulfur-/L-Cysteine starvation | -3.22 | 0.0397 |
| b1729 | *tcyP* | sulfur-/L-Cysteine starvation | -3.20 | 0.0401 |
| b1287 | *yciW* | sulfur-/L-Cysteine starvation | -3.12 | 0.0447 |

**Additional file 1: table S10:** Table showing accession numbers, gene names, features, logarithmic fold changes (logFC) and p-values of all differentially expressed genes (DEGs) of W3110_pCYS_m with p-values <0.05. LogFC and logCPM were calculated by dividing values of the later generation population (LGP) by values of the early generation population (EGP).

| **Accession** | **Gene** | **Feature** | **logFC** | **P-Value** |
| --- | --- | --- | --- | --- |
| b0366 | *tauB* | sulfur-/ L-cysteine starvation | -5.821272583 | 2.98E-05 |
| b0367 | *tauC* | sulfur-/ L-cysteine starvation | -5.082104853 | 0.000155955 |
| b0365 | *tauA* | sulfur-/ L-cysteine starvation | -4.903006691 | 0.000213135 |
| b0936 | *ssuA* | sulfur-/ L-cysteine starvation | -4.784398761 | 0.000363555 |
| b0935 | *ssuD* | sulfur-/ L-cysteine starvation | -4.424511323 | 0.00064772 |
| b1493 | *gadB* | acid resistance | -4.393691919 | 0.000650756 |
| b4470 | *cyuA* | l-Cysteine degradation | 4.20665212 | 0.00099003 |
| b1492 | *gadC* | acid resistance | -4.200031995 | 0.001002402 |
| b0937 | *ssuE* | sulfur-/ L-cysteine starvation | -4.304585235 | 0.001055832 |
| b0368 | *tauD* | sulfur-/ L-cysteine starvation | -4.130291126 | 0.00123138 |
| b0450 | *glnK* | nitrogen starvation | -3.950577269 | 0.001776567 |
| b3707 | *tnaC* | transcriptional attenuation | -4.18533155 | 0.002304927 |
| b0934 | *ssuC* | sulfur-/ L-cysteine starvation | -3.810239622 | 0.002799372 |
| b0451 | *amtB* | nitrogen starvation | -3.678782354 | 0.003164359 |
| b1489 | *dosP* | oxygen sensing | -3.693422741 | 0.003226504 |
| b3708 | *tnaA* | tryptophanases | -3.633665908 | 0.003575459 |
| b2750 | *cysC* | sulfur-/ L-cysteine starvation | -3.599451267 | 0.003912051 |
| b2298 | *yfcC* | unknown function | 3.592554082 | 0.004078273 |
| b1488 | *ddpX* | nitrogen starvation | -3.623180338 | 0.004573246 |
| b1748 | *astC* | L-arginine catabolysis | -3.518503904 | 0.004670165 |
| b1988 | *nac* | nitrogen starvation | -3.434444053 | 0.005431418 |
| b3917 | *sbp* | sulfur-/ L-cysteine starvation | -3.435172087 | 0.005444333 |
| b1467 | *narY* | nitrate assimilation | -3.438555719 | 0.005601404 |
| b3511 | *hdeD* | acid resistance | -3.361747128 | 0.006279399 |
| b1466 | *narW* | nitrate assimilation | -3.391764903 | 0.006610653 |
| b1747 | *astA* | L-arginine catabolysis | -3.327739224 | 0.006992027 |
| b1012 | *rutA* | pyrimidin degradation | -3.421121875 | 0.007184279 |
| b1182 | *hlyE* | hemolysis | 3.512051653 | 0.00775016 |
| b3517 | *gadA* | acid resistance | -3.221148839 | 0.008425411 |
| b2751 | *cysN* | sulfur-/ L-cysteine starvation | -3.206799828 | 0.008634876 |
| b1468 | *narZ* | nitrate assimilation | -3.147760624 | 0.009868409 |
| b4314 | *fimA* | cell adhesion | -3.006357837 | 0.013196354 |
| b3671 | *ilvB* | BCAA biosynthesis | -2.994222798 | 0.013343519 |
| b2763 | *cysI* | sulfur-/ L-cysteine starvation | -2.980844596 | 0.013714836 |
| b3509 | *hdeB* | acid resistance | -2.947653324 | 0.014698199 |
| b3268 | *yhdW* | unknown function | -2.936914268 | 0.015223895 |
| b1038 | *csgF* | curli secretion | -2.979648511 | 0.015299404 |
| b1487 | *ddpA* | nitrogen starvation | -2.921872385 | 0.01666648 |
| b1541 | *ydfZ* | unknown function | 2.904012063 | 0.016843664 |
| b1465 | *narV* | nitrate assimilation | -2.889579459 | 0.017708658 |
| b3514 | *mdtF* | multidrug efflux | -2.848462657 | 0.018047176 |
| b3110 | *cyuP* | l-Cysteine degradation | 2.821287725 | 0.018927267 |
| b1987 | *cbI* | sulfur-/ L-cysteine starvation | -2.823628515 | 0.019316605 |
| b1746 | *astD* | L-arginine catabolysis | -2.817034764 | 0.019391912 |
| b2111 | *yehD* | biofilm formation | 3.261253189 | 0.019492368 |
| b3510 | *hdeA* | acid resistance | -2.791811406 | 0.020018914 |
| b2422 | *cysA* | sulfur-/ L-cysteine starvation | -2.773039444 | 0.020788403 |
| b0992 | *yccM* | unknown function | 2.841407281 | 0.02087771 |
| b2752 | *cysD* | sulfur-/ L-cysteine starvation | -2.728603797 | 0.02271106 |
| b4072 | *nrfC* | anaerobic respiration | 2.933523439 | 0.022806289 |
| b2343 | *yfcZ* | unknown function | 2.714277519 | 0.023304408 |
| b4070 | *nrfA* | anaerobic respiration | 2.73552111 | 0.023744641 |
| b2423 | *cysW* | sulfur-/ L-cysteine starvation | -2.71382996 | 0.023758522 |
| b0933 | *ssuB* | sulfur-/ L-cysteine starvation | -2.726988746 | 0.024362651 |
| b0896 | *dmsC* | anaerobic respiration | 2.659026564 | 0.026118345 |
| b2110 | *yehC* | biofilm formation | 3.86463591 | 0.026778193 |
| b4116 | *adiY* | transcriptional activation | 2.619087737 | 0.028356012 |
| b1745 | *astB* | L-arginine catabolysis | -2.625927022 | 0.028460359 |
| b1224 | *narG* | nitrate assimilation | -2.598496723 | 0.029222593 |
| b3158 | *ubiU* | ubiquinone biosynthesis | 2.611203373 | 0.029286652 |
| b2310 | *argT* | nitrogen starvation | -2.581918264 | 0.030323738 |
| b2764 | *cysJ* | sulfur-/ L-cysteine starvation | -2.569263788 | 0.030819373 |
| b3670 | *ilvN* | BCAA biosynthesis | -2.56428254 | 0.031168241 |
| b1443 | *ydcV* | unknown function | -2.590221267 | 0.031564249 |
| b3269 | *ydhX* | unknown function | -2.595507339 | 0.032143511 |
| b1674 | *ydhY* | unknown function | 2.640124724 | 0.032660893 |
| b0895 | *dmsB* | anaerobic respiration | 2.538107989 | 0.033062582 |
| b2203 | *napB* | anaerobic respiration | 2.590548108 | 0.03328397 |
| b1490 | *dosC* | oxygen sensing | -2.534568305 | 0.033441933 |
| b4245 | *pyrB* | pyrimidin degradation | -2.524023685 | 0.033568073 |
| b3369 | *yhfL* | unknown function | 3.100367778 | 0.033702782 |
| b1011 | *rutB* | pyrimidin degradation | -2.696697026 | 0.034889092 |
| b2345 | *yfdF* | unknown function | 3.18686013 | 0.03551058 |
| b2208 | *napF* | anaerobic respiration | 2.536604392 | 0.035939355 |
| b0991 | *ymcE* | unknown function | 3.068757409 | 0.036027242 |
| b3073 | *patA* | l-lysin degradation | -2.459327192 | 0.03821933 |
| b1731 | *cedA* | cell division regulation | 2.681966451 | 0.038563357 |
| b2292 | *yfbS* | unknown function | 2.451566606 | 0.038713109 |
| b2996 | *hybA* | anaerobic respiration | 2.459889195 | 0.038806123 |
| b0990 | *cspG* | cold shock response | 2.50706977 | 0.03895797 |
| b0894 | *dmsA* | anaerobic respiration | 2.444091285 | 0.039041251 |
| b1287 | *yciW* | hyperosmotic stress | -2.448737859 | 0.039274106 |
| b2676 | *nrdF* | pyrimidin degradation | -2.493438134 | 0.03953287 |
| b1675 | *fumA* | anaerobic respiration | 2.545337693 | 0.039793075 |
| b1025 | *dgcT* | biofilm formation | 2.549287518 | 0.039812429 |
| b1441 | *ydcT* | unknown function | -2.474246634 | 0.040116258 |
| b3709 | *tnaB* | tryptophanases | -2.607284958 | 0.040244207 |
| b2201 | *ccmA* | cytochrome c maturation | 2.44516171 | 0.040712435 |
| b2727 | *hypB* | protein maturation | 2.423184881 | 0.041180646 |
| b4244 | *pyrI* | pyrimidin degradation | -2.40898976 | 0.041669821 |
| b0621 | *dcuC* | succinat efflux | 2.422663786 | 0.041722113 |
| b2202 | *napC* | anaerobic respiration | 2.422513419 | 0.041909772 |
| b0036 | *caiD* | L-carnitine degradation | 2.512422952 | 0.043274337 |
| b2997 | *hybO* | anaerobic respiration | 2.38619037 | 0.043938516 |
| b4606 | *ypfM* | unknown function | 2.496316042 | 0.044010761 |
| b1014 | *putA* | L-proline degradation | -2.371242798 | 0.044714409 |
| b2749 | *ygbE* | unknown function | -2.397245869 | 0.045600936 |
| b3508 | *yhiD* | unknown function | -2.371373692 | 0.046274455 |
| b3513 | *gadW* | acid resistance | -2.354682174 | 0.046389608 |
| b4713 | *agrB* | small regulatory RNA | 2.899398595 | 0.047659431 |
| b0618 | *citC* | citrate lyase | 2.786062243 | 0.047944746 |
| b1039 | *csgE* | curli secretion | -2.386568791 | 0.047956706 |
| b4517 | *gnsA* | unknown function | 2.435844309 | 0.048711147 |
| b1744 | *astE* | L-arginine catabolysis | -2.36011251 | 0.049168527 |
| b2150 | *mglB* | chemotaxis | -2.348907891 | 0.049424165 |

**Additional file 1: table S11:** Table showing accession numbers, gene names, features, logarithmic fold changes (logFC) and p-values of all differentially expressed genes (DEGs) of MDS42_pCYS_m with p-values <0.05. LogFC and logCPM were calculated by dividing values of the later generation population (LGP) by values of the early generation population (EGP).

| **Accession** | **Gene** | **Feature** | **logFC** | **P-Value** |
| --- | --- | --- | --- | --- |
| gene-b4668 | *ibsB* | unknown function | -2.17 | 0.022 |
| gene-b3643 | *rph* | rRNA 3'-end and tRNA processing | -4.98 | 0.027 |
| gene-b1539 | *ydfG* | uracil catabolism | 3.04 | 0.028 |
| gene-b1137 | *ymfD* | unknown function | 2.14 | 0.028 |
| gene-b2848 | *yqeJ* | unknown function | 1.37 | 0.038 |
| gene-b0150 | *yceK* | iron starvation | 4.59 | 0.039 |
| gene-b2724 | *hycB* | glucose catabolism | -1.33 | 0.039 |
| gene-b2481 | *hyfA* | alternative sugar utilizing pathways | 1.22 | 0.043 |
| gene-b3900 | *frvA* | glucose catabolism | -1.40 | 0.044 |
| gene-b4268 | *idnK* | alternative sugar utilizing pathways | 1.12 | 0.047 |
| gene-b0532 | *sfmD* | pilus organization | -1.13 | 0.047 |
| gene-b0040 | *caiT* | nitrogen starvation | -1.13 | 0.048 |
| gene-b4181 | *yjfl* | unknown function | 1.24 | 0.048 |
| gene-b4254 | *argI* | nitrogen starvation | -1.02 | 0.049 |

**Additional file1: table S12:** Values of calculated plasmid DNA contents extracted from early and late generation populations (EGP, LGP). Plasmid DNA was extracted from 10 ml cultures and eluted in 50 µl each. Cell dry weights were extrapolated with the factor of 0.33 g/L/OD_600= 1.0_ for *E. coli* K-12 MG1655 cells according to Sauer et al (2).

| Plasmid | **pCYS_m** | | | | **pCYS_i** | | | | **pCYS** | | | |
| --- | --- | --- | --- | --- | --- | --- | --- | --- | --- | --- | --- | --- |
| Strain | **W3110** | | **MDS42** | | **W3110** | | **MDS42** | | **W3110** | | **MDS42** | |
| Population | **EGP** | **LGP** | **EGP** | **LGP** | **EGP** | **LGP** | **EGP** | **LGP** | **EGP** | **LGP** | **EGP** | **LGP** |
| OD_600_ | 4.3 | 4.8 | 4.6 | 4.7 | 4.4 | 4.6 | 4.8 | 4.4 | 4.5 | 4.3 | 4 | 4.4 |
| Cell Dry mass [µg] | 1.42E+04 | 1.58E+04 | 1.52E+04 | 1.55E+04 | 1.45E+04 | 1.52E+04 | 1.58E+04 | 1.45E+04 | 1.49E+04 | 1.42E+04 | 1.32E+04 | 1.45E+04 |
| pDNA [ng/µl] | **96** | **112** | **52** | **38** | **103** | **123** | **44** | **47** | **67** | **75** | **51** | **62** |
| pDNA mass [µg] | 4.82 | 5.63 | 2.62 | 1.9 | 5.15 | 6.18 | 2.23 | 2.36 | 3.4 | 3.8 | 2.6 | 3.13 |
| µg pDNA/ µg cells | 3.40E-04 | 3.56E-04 | 1.73E-04 | 1.23E-04 | 3.55E-04 | 4.08E-04 | 1.40E-04 | 1.63E-04 | 2.29E-04 | 2.65E-04 | 1.94E-04 | 2.16E-04 |

**Additional file 1: table S13:** Single-nucleotide polymorphism (SNP) variant table. The SNP calling was done using VarSCan2 (3). Allele frequency cut-off used for variant calling was 1%. For each sample, the following variant summary is provided: Strain, plasmid, population. Additionally, POS: Position at which the variant was observed, BB/ORF: Location affected by the mutation (BB: backbone, ORF: open reading frame), REF: Reference base, ALT: Alternative base, Allele Freq: Variant allele frequency in percentage, Alt Depth: Depth of variant-supporting bases, total depth: Depth of variant-supporting bases and reference-supporting bases. Mutations which showed allele-frequencies >95% were assumed to be originally present in the plasmids.

| Strain | Plasmid | Population | POS | BB /ORF | REF | ALT | Allele-Freq | Alt_Depth | Total_Depth |
| --- | --- | --- | --- | --- | --- | --- | --- | --- | --- |
| MDS42 | pCYS | EGP  LGP | 3057 | BB | A | C | 55.14 | 3425 | 206153 |
|  |  |  | 3057 | BB | A | C | 54.19 | 4131 | 248335 |
|  | pCYS_i | EGP | - | - | - | - | - | - | - |
|  |  | LGP | - | - | - | - | - | - | - |
|  | pCYS_m | EGP | 1253 | BB | G | GT | 95.65 | 140272 | 134362 |
|  |  |  | 1397 | BB | A | T | 1.07 | 1162 | 108962 |
|  |  |  | 2523 | BB | C | A | 3.4 | 3818 | 112358 |
|  |  |  | 2637 | BB | G | A | 99.93 | 151469 | 151574 |
|  |  |  | 2625 | BB | A | T | 99.87 | 148937 | 149131 |
|  |  | LGP | 2523 | BB | C | A | 3.38 | 4360 | 129097 |
|  |  |  | 2637 | BB | G | A | 99.98 | 179578 | 179647 |
|  |  |  | 2625 | BB | A | T | 99.89 | 170523 | 170718 |
|  |  |  | 2645 | BB | A | C | 99.98 | 179578 | 179647 |
|  |  |  | 1253 | BB | G | GT | 95.83 | 157198 | 152298 |
| W3110 | pCYS | EGP | 3057 | BB | A | C | 54.09 | 3040 | 171653 |
|  |  | LGP | 3057 | BB | A | C | 54.66 | 2446 | 140778 |
|  | pCYS_i | EGP | - | - | - | - | - | - | - |
|  |  | LGP | - | - | - | - | - | - | - |
|  | pCYS_m | EGP | 1253 | BB | G | GT | 95.3 | 105873 | 93750 |
|  |  |  | 2523 | BB | C | A | 3.89 | 3147 | 80897 |
|  |  |  | 2625 | BB | A | T | 99.91 | 108989 | 109092 |
|  |  |  | 2637 | BB | G | A | 99.95 | 169794 | 169866 |
|  |  |  | 2645 | BB | A | C | 99.96 | 113604 | 113669 |
|  |  | LGP | 1397 | BB | A | T | 1.11 | 1308 | 117871 |
|  |  |  | 2523 | BB | C | A | 3.65 | 4455 | 122235 |
|  |  |  | 2645 | BB | A | C | 99.97 | 169794 | 169866 |
|  |  |  | 2625 | BB | A | T | 99.9 | 161681 | 161857 |
|  |  |  | 1253 | BB | G | GT | 95.5 | 153242 | 144568 |


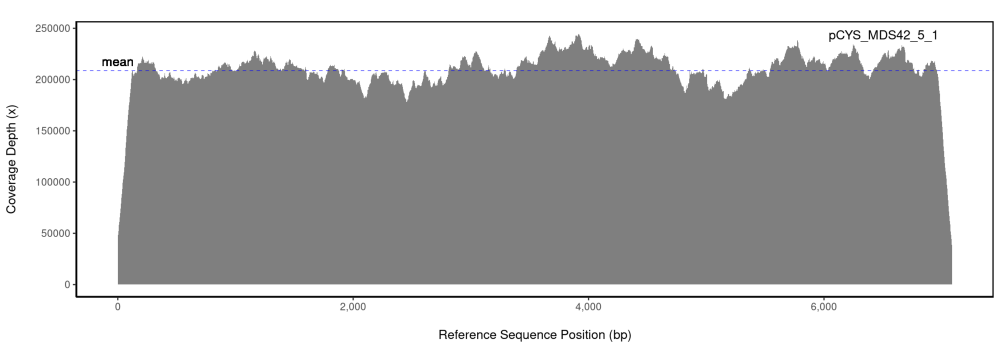


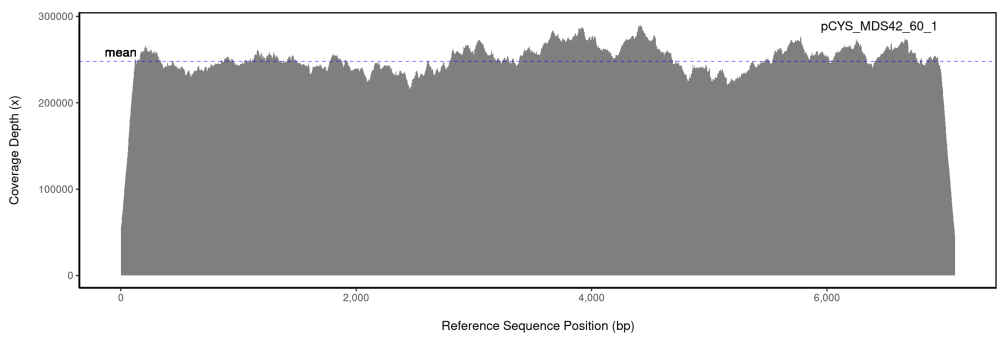


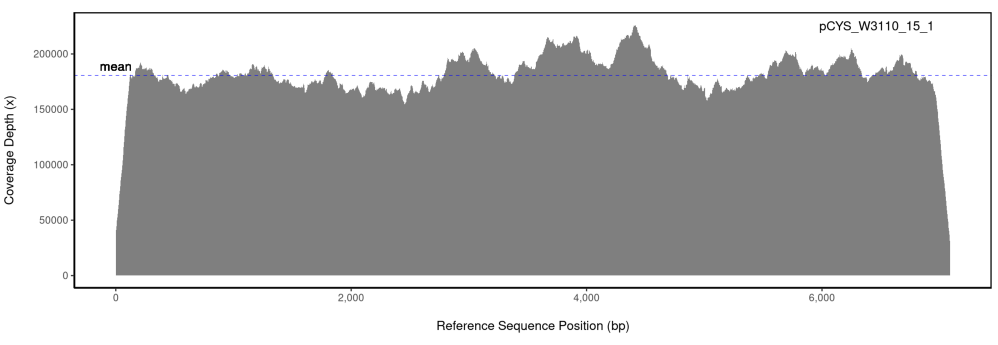

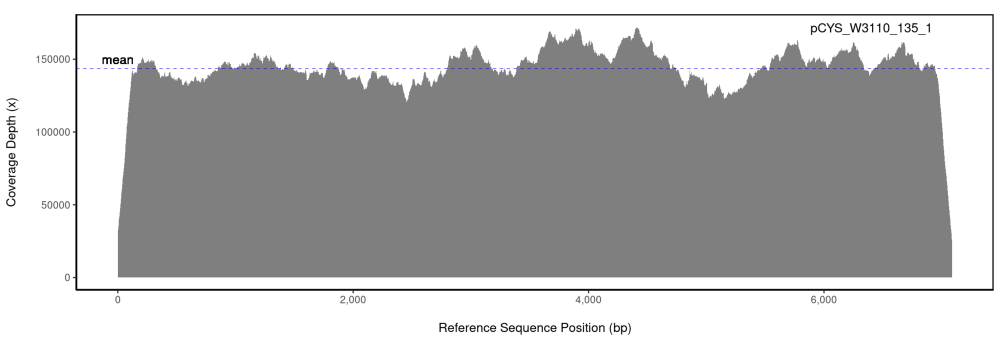


**Additional file 1: figure S2:** Per base coverage depths (x) of sequenced pCYS extracted from early and late generation populations (EGPs and LGPs) of *E. coli* W3110 and MDS42. Sequencing was conducted with Illumina Novaseq paired end 2x150bp.


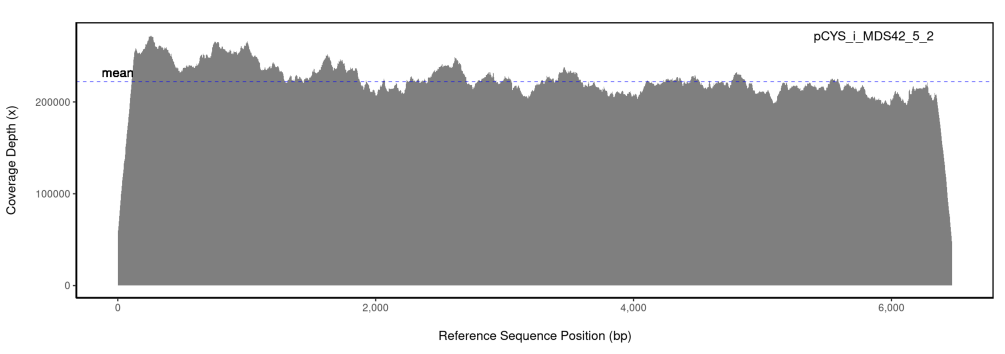


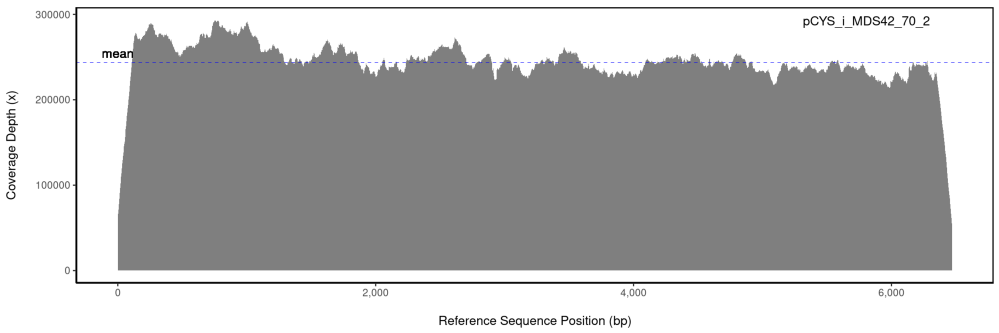


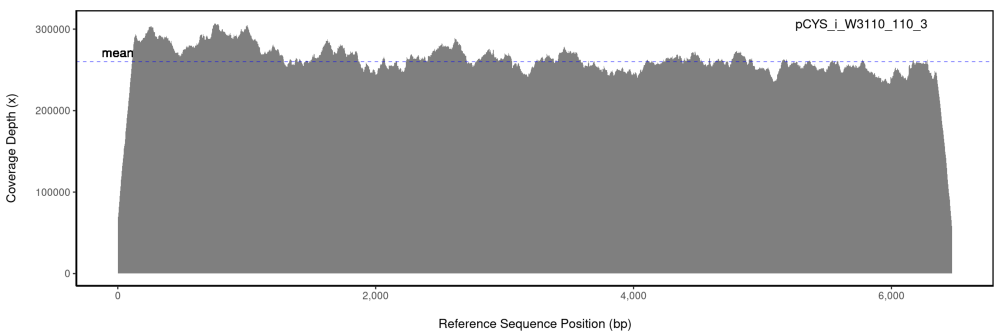


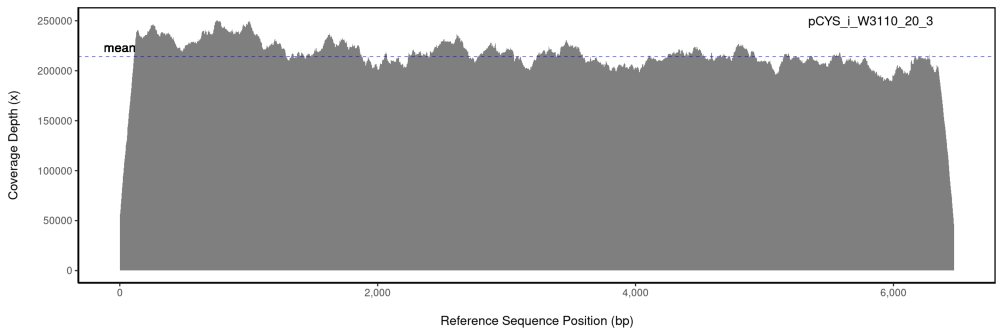


**Additional file 1: figure S3:** Per base coverage depths (x) of sequenced pCYS_i extracted from early and late generation populations (EGPs and LGPs) of *E. coli* W3110 and MDS42. Sequencing was conducted with Illumina Novaseq paired end 2x150bp.


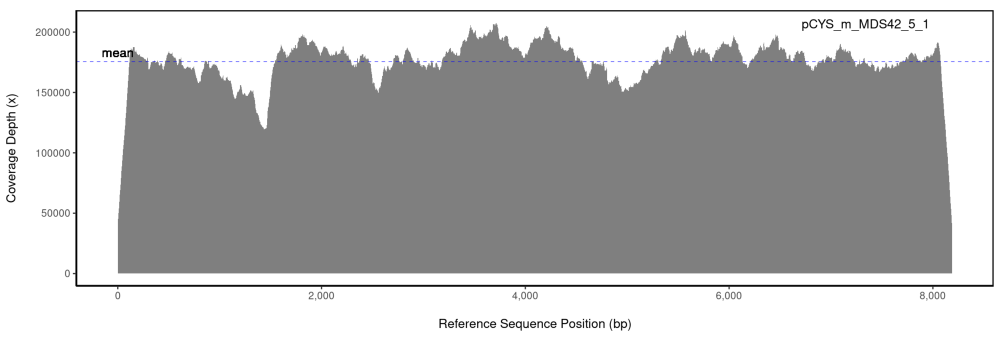


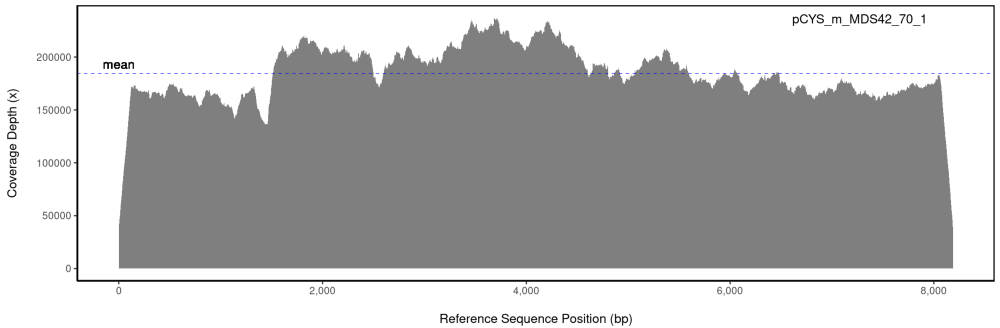


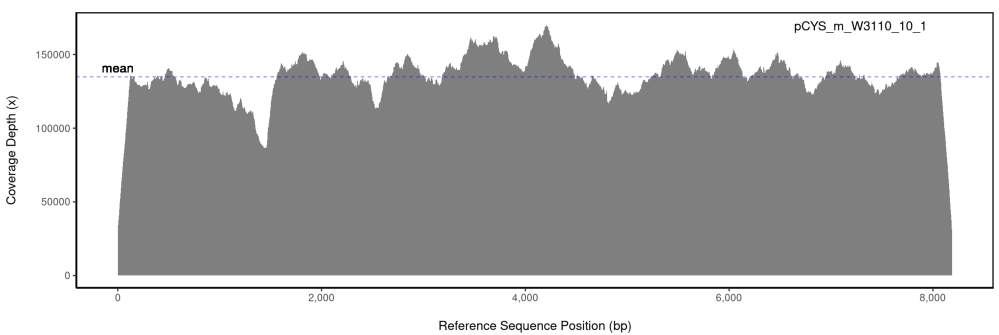


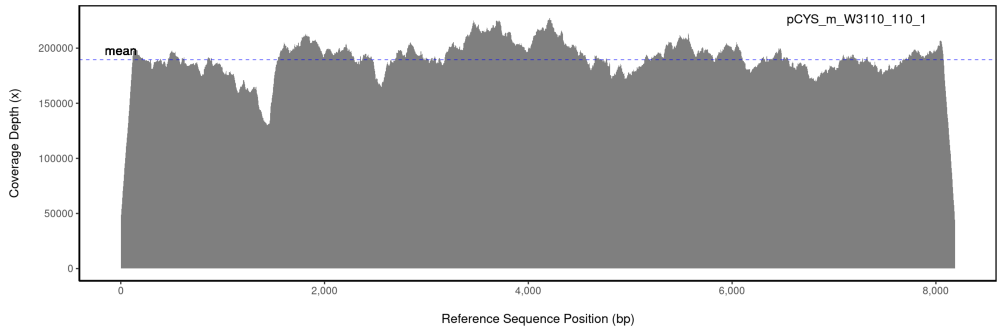


**Additional file1: figure S4:** Per base coverage depths (x) of sequenced pCYS_m extracted from early and late generation populations (EGPs and LGPs) of *E. coli* W3110 and MDS42. Sequencing was conducted with Illumina Novaseq paired end 2x150bp.

1. Gaitonde MK. A spectrophotometric method for the direct determination of Cysteine in the presence of other naturally occuring amino acids. Biochem J. 1967;104:627-33.

2. Sauer U. Metabolic flux ratio analysis of genetic and environmental modulations of Escherichia coli central carbon metabolism. AMS for Microbiol 1999;181(21):6679-88.

3. Koboldt DC, Chen K, Wylie T, Larson DE, McLellan MD, Mardis ER, et al. VarScan: variant detection in massively parallel sequencing of individual and pooled samples. Bioinformatics. 2009;25(17):2283-5.
